# Temporal association between invasive procedures and infective endocarditis

**DOI:** 10.1136/heartjnl-2022-321519

**Published:** 2022-09-22

**Authors:** Martin H Thornhill, Annabel Crum, Richard Campbell, Tony Stone, Ellen C Lee, Mike Bradburn, Veronica Fibisan, Mark Dayer, Bernard D Prendergast, Peter Lockhart, Larry Baddour, Jon Nicoll

**Affiliations:** 1 Department of Oral & Maxillofacial Medicine, Surgery and Pathology, The University of Sheffield School of Clinical Dentistry, Sheffield, UK; 2 Department of Oral Medicine/Oral & Maxillofacial Surgery, Carolinas Medical Center, Charlotte, North Carolina, USA; 3 The University of Sheffield School of Health and Related Research, Sheffield, UK; 4 CTRU, University of Sheffield School of Health and Related Research, Sheffield, UK; 5 Department of Cardiology, Somerset Foundation Trust, Taunton, Somerset, UK; 6 Department of Cardiology, St Thomas’ Hospital, London, UK; 7 Departments of Medicine and Cardiovascular Disease, Mayo Clinic College of Medicine and Science, Rochester, Minnesota, USA

**Keywords:** endocarditis

## Abstract

**Objective:**

Antibiotic prophylaxis has been recommended for patients at increased risk of infective endocarditis (IE) undergoing specific invasive procedures (IPs) despite a lack of data supporting its use. Therefore, antibiotic prophylaxis recommendations ceased in the mid-2000s for all but those at high IE risk undergoing invasive dental procedures. We aimed to quantify any association between IPs and IE.

**Methods:**

All 14 731 IE hospital admissions in England between April 2010 and March 2016 were identified from national admissions data, and medical records were searched for IP performed during the 15-month period before IE admission. We compared the incidence of IP during the 3 months immediately before IE admission (case period) with the incidence during the preceding 12 months (control period) to determine whether the odds of developing IE were increased in the 3 months after certain IP.

**Results:**

The odds of IE were increased following permanent pacemaker and defibrillator implantation (OR 1.54, 95% CI 1.27 to 1.85, p<0.001), extractions/surgical tooth removal (OR 2.14, 95% CI 1.22 to 3.76, p=0.047), upper (OR 1.58, 95% CI 1.34 to 1.85, p<0.001) and lower gastrointestinal endoscopy (OR 1.66, 95% CI 1.35 to 2.04, p<0.001) and bone marrow biopsy (OR 1.76, 95% CI 1.16 to 2.69, p=0.039). Using an alternative analysis, bronchoscopy (OR 1.33, 95% CI 1.06 to 1.68, p=0.049) and blood transfusions/red cell/plasma exchange (OR 1.2, 95% CI 1.07 to 1.35, p=0.012) were also associated with IE.

**Conclusions:**

This study identifies a significant association between specific IPs (permanent pacemaker and defibrillator implantation, dental extraction, gastrointestinal endoscopy and bronchoscopy) and subsequent IE that warrants re-evaluation of current antibiotic prophylaxis recommendations to prevent IE in high IE risk individuals.

**WHAT IS ALREADY KNOWN ON THIS TOPIC**

- Antibiotic prophylaxis (AP) was recommended before various invasive procedures (IPs) to prevent infective endocarditis (IE), but in the mid-2000s, this practice was stopped (except for invasive dental procedures in those at high risk outside the UK), due to an absence of evidence associating these procedures with IE; in the UK, AP stopped altogether. Since then, there has been a significant increase in IE incidence in the UK and the rest of Europe.

**WHAT THIS STUDY ADDS**

- This study investigated any temporal association between IPs and subsequent IE in England and identified a significant association with IE following implantation of cardiac pacemakers/defibrillators (CIEDs), dental extractions/surgical tooth removal, upper and lower gastrointestinal endoscopy, and bronchoscopy, all previously recommended for AP.

**HOW THIS STUDY MIGHT AFFECT RESEARCH, PRACTICE OR POLICY**

- These findings provide evidence to warrant a re-evaluation of current AP recommendations for IE prevention in those at high IE-risk, particularly with regard to implantation of CIEDs, gastrointestinal endoscopy, dental extractions (in the UK) and bronchoscopy.

## Introduction

Infective endocarditis (IE) incidence has increased significantly in the last decade in the UK^1^ and the rest of Europe.^2^ Responsible factors could include an ageing population, increased intracardiac device use (pacemakers, implantable cardioverter-defibrillators, surgical and transcatheter heart valves), vascular interventions (including haemodialysis), injection drug use, greater IE awareness and access to investigations (especially echocardiography), and changes in IE prevention guidelines.^1^

IE has devastating consequences, and prevention has been the focus of guidelines. Previous UK, European and US guidelines recommended AP for moderate or high IE risk patients undergoing various invasive procedures (IPs), including invasive-dental procedures (IDPs) (online supplemental appendix table S1). With the possible exception of IDPs, however, there is scant evidence linking IPs to IE or evidence that AP prevents IE.^3^ This, and concerns about adverse drug reactions and the development of antibiotic resistance, led the American Heart Association (AHA)^4^ and European Society for Cardiology (ESC)^5^ to recommend restricting AP use to IDPs in those at high IE risk and the UK National Institute for Health and Care Excellence (NICE) to recommend the complete cessation of AP to prevent IE.^6^

10.1136/heartjnl-2022-321519.supp1 Supplementary data

This study aimed to investigate any association between specific IPs and subsequent IE in England using a case-crossover methodology during a period when AP prevention of IE was not recommended.

## Methods

### IE admissions and IE risk stratification

All hospital admissions in England are recorded in the Hospital Episode Statistics (HES) database. With UK National Research Ethics Service approval (17/SC/0371) and Confidentiality Advisory Group approval, this resource was used to identify all IE admissions between 1 April 2010 and 31 March 2016. An admission was defined as a single continuous hospital stay (which could comprise several consultant episodes), where an International Classification of Diseases 10th Revision (ICD-10) primary or secondary diagnosis code I33.0, I33.9, I39.0, I39.1, I39.2, I39.3, I39.4 or I39.8, or a primary diagnosis code I38.X, was used for any consultant episode. Patients discharged alive with a <3 day length of hospital stay or elective admission were excluded.^7^ This study is reported according to Strengthening the Reporting of Observational Studies in Epidemiology guidelines.

Each patient’s HES record was retrieved from 1 January 2000. To stratify individuals into high, moderate or low/unknown risk of IE (box 1), records were searched for ICD-10 diagnosis or Office of Population Censuses and Surveys Classification of Surgical Operations and Procedures Revision 4 (OPCS-4) procedure codes occurring before IE admission that placed them into these categories based on ESC and AHA guidelines (box 1, online supplemental tables S1 and 2).^4 5^

Box 1 **Cardiac conditions used to classify individuals as being at high or moderate infective endocarditis (IE) risk**

**High IE risk.**

Previous history of IE.

Presence of prosthetic heart valve (including transcatheter valves).

Prosthetic material used for valve repair (including annuloplasty and transcatheter valve procedures).

Unrepaired cyanotic congenital heart disease.

Congenital heart disease treated with palliative shunts or conduits.

Congenital heart defect repaired with surgical or transcatheter technique using prosthetic material or device (first 6 months postprocedure only).

**Moderate IE risk**

Rheumatic heart disease.

Non-rheumatic valve disease (including mitral valve prolapse).

Congenital valve anomalies (including aortic stenosis).

Hypertrophic cardiomyopathy.

**Notes:** adapted from the European Society of Cardiology and American Heart Association guidelines.^7 8 11 12^ More extensive details of all diagnoses and procedures (including relevant ICD-10 diagnosis or OPCS-4 procedure codes) included in the definition of those at high or moderate IE risk are provided in online supplemental tables S2 and S3).

New IE admissions were distinguished from readmissions by only accepting IE admissions >180 days apart. Consistent with the guidelines, individuals with congenital heart disease completely repaired with prosthetic material or a device were considered high-risk for IE for 6 months after the procedure and then considered low risk. Individuals not identified as moderate or high risk were considered at low/unknown risk of IE.

### Patient and public involvement

Patients were not directly involved in this study.

### Invasive procedures

Each patient’s record was searched for OPCS-4 IP codes of interest (online supplemental tables S4 and S5) for each 30-day period over the 15 months before IE admission (ie, between 1 January 2009 and 31 March 2016). IPs of interest were those previously recommended for AP in the 2004 British Cardiac Society and 2006 British Society for Antimicrobial Chemotherapy guidance or identified as associated with an increased risk of IE in a recent Swedish study (online supplemental table S1). To avoid the possibility of reverse causation (procedures being performed as part of the investigation or management of IE), we excluded procedures undertaken during the IE admission. Because some cardiac IPs, for example, coronary artery bypass grafting, may be performed simultaneously with procedures such as valve replacement or repair, we only included them when they occurred alone. Although an association has been reported between dialysis and IE,^8^ the case-crossover methodology is inappropriate for a procedure performed with such regularity, and dialysis was excluded from the study. To ensure we counted the number of individuals exposed to each procedure each month (rather than the number of procedures), we counted the first procedure of each type performed on each individual each month.

Restricting IP data to 1 January 2009 through 31 March 2016, meant all IPs were performed after NICE recommended AP use to prevent IE cease (March 2008)^6^ and before any relaxation of this (April 2016).^9^ Thus, any association between IPs and IE should have been fully exposed.

### Case-crossover study

#### Primary analysis

**Figure 1:**
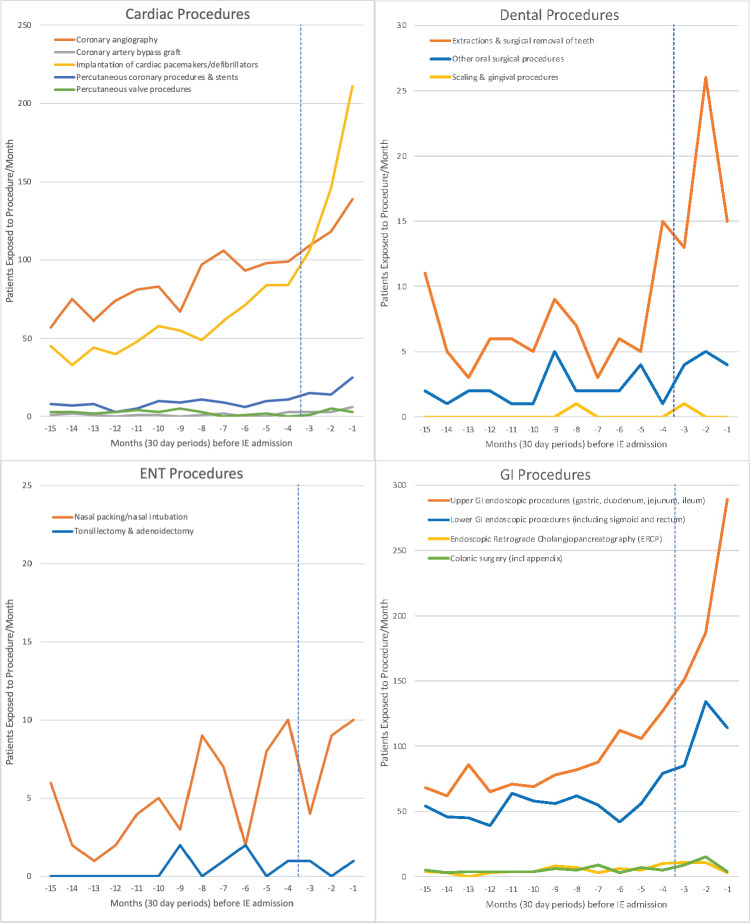
Incidence of different cardiac, dental, ENT and GI invasive procedures over the 15 months before infective endocarditis (IE) hospital admission. Vertical blue dashed line separates case period (months −1 to −3) from control period (months −4 to −15). ENT, ear, nose and throat; GI, gastrointestinal.

**Figure 2:**
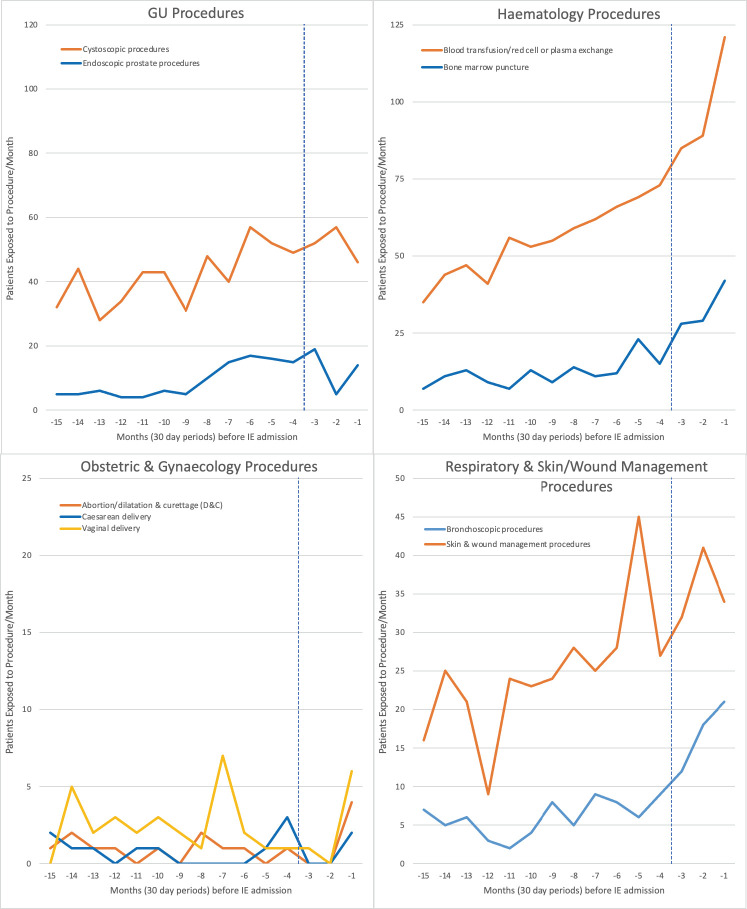
Incidence of different GU, haematology, obstetrics and gynaecology, respiratory and skin/wound management invasive procedures over the 15 months before infective endocarditis (IE) hospital admission. Vertical blue dashed line separates case period (months −1 to −3) from control period (months −4 to −15). GU, genitourinary.

Monthly exposure to IPs was quantified over the 15 months before IE-related hospital admissions to determine any temporal association (figures 1 and 2 and online supplemental figure S1). Using a step model case-crossover analysis for each IP,^10^ we calculated the period-adjusted OR and its 95% CI of that IP having been undertaken during the 3-month case period before IE admission compared with the preceding 12-month control period (months 4–15), using a mixed-effects logistic regression model with the patient as a random effect and a fixed effect step parameter at 3 months. To account for potential temporal bias of increasing numbers of IPs being performed, we also calculated an adjusted OR for each IP using a mixed-effects logistic regression model adjusted for date of IE admission (see online supplemental appendix methods). Statistical analyses were performed in Stata V.17, using core packages, and all p values were corrected upwards for multiple comparisons using the Benjamini-Hochberg method.^11^

#### Secondary analysis

Sensitivity analyses were performed using 4-month and 6-month case periods (online supplemental tables S7 and S8), and an alternative ‘hinge-model’ case-crossover analysis was performed (see online supplemental appendix), in which instead of fitting a step change at 3 months, we fitted a change in the time trend at 3 months before admission (a linear term for months -3 to -1).

#### Attributable risk

**Figure 3:**
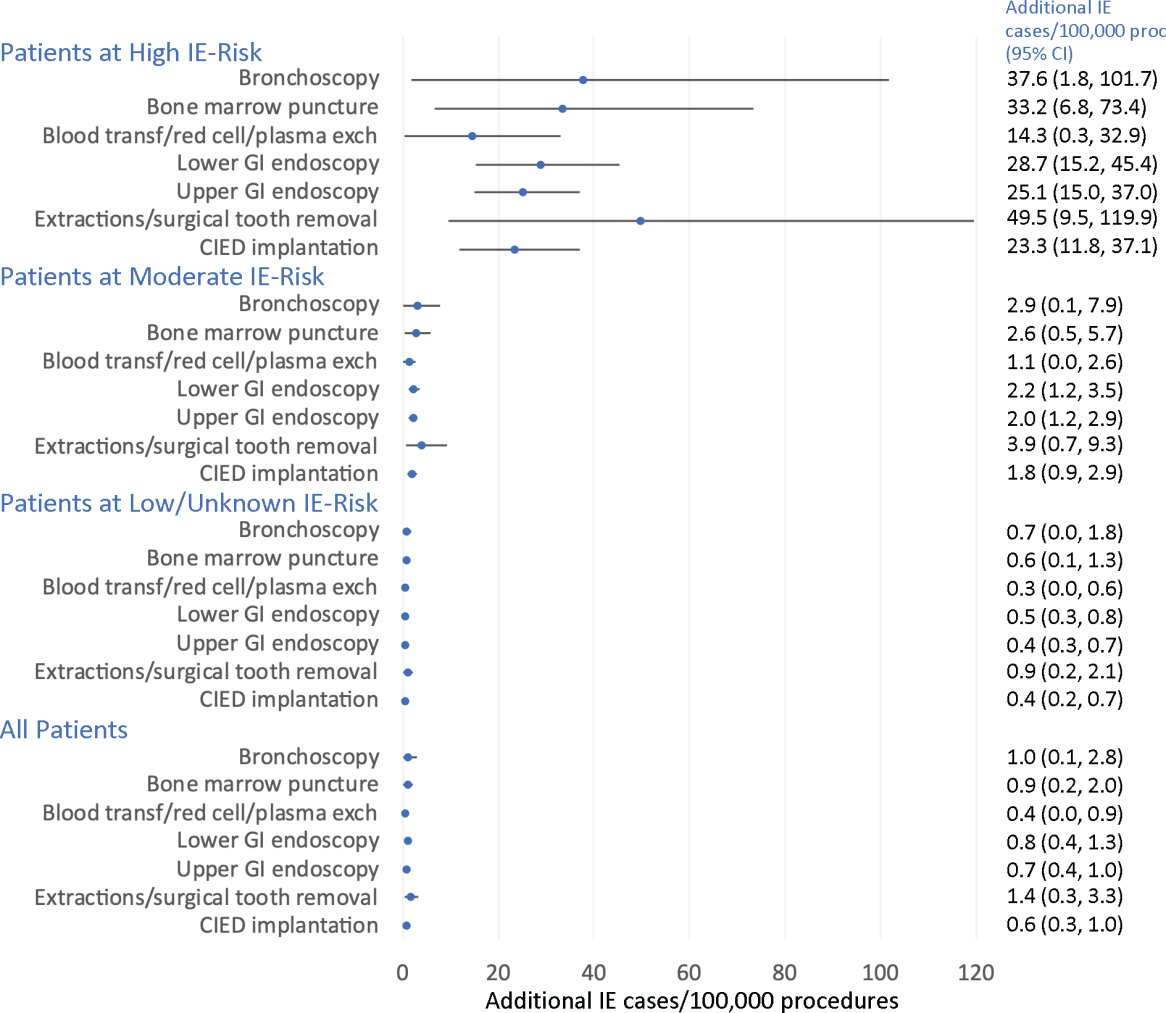
Attributable risk – the predicted additional IE cases per 100 000 procedures by patient risk group. The attributable risk is presented for IPS with a significant positive temporal association with subsequent IE and is expressed as the predicted additional number of IE cases per 100 000 procedures. The population at risk was estimated using the population of England during the middle year of the study (2012–2023) and estimates for the proportion of the population at high, moderate or low/unknown risk. Baseline risk was calculated as the average 3 monthly risk of being subject to each procedure for each population over the study period (March 2010–December 2015, excluding the last 3 months of data). The attributable risk was calculated by multiplying the baseline risk with the adjusted or estimate from table 1. CIED, cardiac implantable electronic devises; exch, exchange; GI, gastrointestinal; IE, infective endocarditis; IPS, invasive procedures; transf, transfusion.

Attributable risk (or absolute risk increase) was defined as the additional number of IE cases per 100 000 procedures) and was estimated for IPs with a significant positive association with IE. The background IE incidence was estimated by dividing the total number of IE cases identified during the study by (duration of study × 53.4937 million), the latter being the Office of National Statistics figure for the population of England during the middle year of the study (2012). The attributable risk per 100 000 procedures was then calculated as=100 000 × background IE incidence × (adjusted OR-1)/4, where the adjusted OR from the primary analysis in table 1 and was used to approximate the relative risk for the 3-month case period, and the denominator (4) reflected the case period was one-quarter of a year. The attributable risk was estimated separately for patients at high, moderate or low/unknown risk using previously published prevalence data for these populations (figure 3).^12 13^

**Table 1:** Case-crossover analysis comparing the incidence of invasive procedures (IPs) in the 3-month case period and the preceding 12-month control period for 14 731 patients admitted with IE

Invasive procedures (ISPs)	Case period (3 m)		Control period (12 m)		Unadjusted step model†		Adjusted step model‡			Adjusted hinge model§		
	Total proc*	Proc/m*	Total proc*	Proc/m*	OR	95% CI	OR	95% CI	P value	OR	95% CI	P value
Cardiac procedures												
Coronary angiography	366	122	991	82.6	1.48	1.31 to 1.67	1.05	0.88 to 1.25	0.776	1.04	0.97 to 1.12	0.403
Coronary artery bypass graft (CABG)	12	4	12	1	4	1.80 to 8.91	2.99	0.75 to 11.96	0.253	1.62	0.96 to 2.73	0.132
Percutaneous coronary procedures and stent implantation	54	18	97	8.1	2.25	1.61 to 3.15	1.59	0.94 to 2.68	0.211	1.28	1.03 to 1.58	0.066
Implantation of cardiac pacemakers/defibrillators	463	154.3	672	56	2.81	2.50 to 3.17	1.54	1.27 to 1.85	<0.001	1.29	1.19 to 1.39	<0.001
Percutaneous valve procedures	9	3	29	2.4	1.25	0.59 to 2.66	2.57	0.78 to 8.45	0.278	1.61	0.99 to 2.60	0.115
Dental procedures												
Extractions or surgical removal of teeth	54	18	81	6.8	2.68	1.90 to 3.78	2.14	1.22 to 3.76	0.047	1.27	1.02 to 1.59	0.082
Other oral surgical procedures	13	4.3	25	2.1	2.10	1.07 to 4.12	1.59	0.56 to 4.53	0.590	1.14	0.75 to 1.75	0.687
Scaling and gingival procedures	1	0.3	1	0.1	4	0.25 to 63.92	2.99	0.02 to 363.52	0.753	0.55	0.08 to 4.04	0.611
ENT procedures												
Tonsillectomy and adenoidectomy	2	0.7	6	0.5	1.33	0.27 to 6.61	0.28	0.03 to 2.39	0.472	0.58	0.21 to 1.56	0.376
Nasal packing/nasal intubation	23	7.7	59	4.9	1.60	0.97 to 2.64	0.71	0.35 to 1.44	0.572	0.99	0.73 to 1.33	0.925
GI procedures												
Upper GI endoscopic procedures (gastric, duodenum, jejunum, ileum)	627	209	1014	84.5	2.59	2.34 to 2.87	1.58	1.34 to 1.85	<0.001	1.30	1.22 to 1.39	<0.001
Lower GI endoscopic procedures (including sigmoid and rectum)	333	111	656	54.7	2.07	1.81 to 2.37	1.66	1.35 to 2.04	<0.001	1.23	1.13 to 1.34	<0.001
Colonic surgery (including appendicectomy)	28	9.3	59	4.9	1.9	1.21 to 2.98	1.48	0.74 to 2.95	0.467	1.01	0.76 to 1.35	0.911
Endoscopic retrograde cholangio-pancreatic procedures	25	8.3	57	4.8	1.81	1.12 to 2.94	0.94	0.46 to 1.89	0.853	0.78	0.57 to 1.06	0.198
GU procedures												
Cystoscopy procedures	155	51.7	501	41.8	1.26	1.05 to 1.53	0.92	0.70 to 1.20	0.775	0.94	0.83 to 1.05	0.391
Endoscopic prostate procedures	38	12.7	108	9	1.41	0.97 to 2.05	0.55	0.33 to 0.92	0.084	0.72	0.57 to 0.91	0.019
Haematology procedures												
Blood transfusion/red cell/plasma exchange	295	98.3	660	55	2.84	2.35 to 3.43	1.33	1.01 to 1.76	0.129	1.2	1.07 to 1.35	0.012
Bone marrow puncture	99	33	144	12	2.96	2.27 to 3.87	1.76	1.16 to 2.69	0.039	1.28	1.08 to 1.52	0.018
Obstetric and gynaecological procedures												
Abortion/dilatation and curettage	4	1.3	11	0.9	1.46	0.46 to 4.63	1.69	0.29 to 9.72	0.754	2.07	0.99 to 4.33	0.120
Vaginal delivery	7	2.3	29	2.4	0.97	0.42 to 2.20	0.96	0.31 to 2.98	0.898	1.34	0.83 to 2.15	0.380
Caesarean delivery	2	0.7	10	0.8	0.8	0.18 to 3.65	0.71	0.10 to 5.24	0.805	1.28	0.56 to 2.94	0.639
Respiratory procedures												
Bronchoscopic procedures	51	17	72	6	2.88	2.00 to 4.13	1.87	1.04 to 3.34	0.118	1.33	1.06 to 1.68	0.049
Skin procedures												
Skin and wound management procedures	107	35.7	295	24.6	1.46	1.17 to 1.83	0.92	0.67 to 1.27	0.765	0.96	0.84 to 1.10	0.600

P values in red=significant positive association between the ISP and subsequent IE after Benjamini-Hochberg correction. P values in purple=significant negative association between ISP and subsequent IE after Benjamini-Hochberg correction.

*A maximum of one procedure of each type per patient was counted each month.

†Period-adjusted OR of ISPs in case period (3 months prior to IE admission) compared with the 12 month control period (15 to 4 months prior to IE admission) calculated using a mixed-effects logistic regression model with the patient as the random effect.

‡OR of ISPs in case period (3 months prior to IE admission) compared with control period (15 to 4 months prior to IE admission) calculated using a mixed-effects logistic regression model adjusted for the month (1–15) and date of IE admission (with the patient as the random effect).

§OR of ISPs for each month increase in the case period (3 to 1 months prior to IE admission) compared with control period (15 to 4 months prior to IE admission) calculated using a mixed-effects logistic regression model adjusted for month and date of IE admission (with patient as the random effect).

IE, infective endocarditis; ISPs, invasive procedures; m, month; proc, procedures.

## Results

### Study population demographics

Between 1 April 2010 and 31 March 2016, there were 14 731 IE admissions (mean age 62.3 years, 66.1% male) in England (table 2). Twenty-one per cent occurred in individuals at high IE risk, 17.0% at moderate risk and 61.7% in those at low/unknown risk. Time course studies plotting the monthly incidence of each IP over 15 months before IE admission are shown in figures 1 and 2.

**Table 2:** Clinical characteristics of patients requiring IE admission

Characteristics	Statistics	All IE admissions
Age	N	14 722
	Mean (SD)	62.3 (19.9)
	Median (min, max)	67.0 (0, 103)
Sex	N	14 731
	Male, n (%)	9734 (66.1)
	Female, n (%)	4997 (33.9)
Level of IE risk before admission	N	14 731
	High risk, n (%)	3145 (21.3)
	Moderate risk, n (%)	2503 (17.0)
	Low/unknown risk, n (%)	9083 (61.7)
Reason for high-risk stratification*	Previous IE, n (%)	154 (4.9)
	Previous IE - I38X, n (%)	67 (2.1)
	Replacement heart valve, n (%)	2416 (76.8)
	Repaired heart valve, n (%)	311 (9.9)
	Cyanotic congenital heart disease, n (%)	150 (4.8)
	Repaired congenital heart disease, n (%)	3 (0.1)
	Palliative shunt or conduit, n (%)	37 (1.2)
	Prosthetic heart/ventricular assist device, n (%)	7 (0.2)
Reason for moderate-risk stratification*	Previous rheumatic fever, n (%)	808 (32.3)
	Non-rheumatic valve disease, n (%)	1571 (62.8)
	Congenital valve anomalies, n (%)	58 (2.3)
	Hypertrophic cardiomyopathy, n (%)	66 (2.6)
Admission date	N	14 731
	Min	01 Apr 2010
	Max	28 Mar 2016
Discharge date	N	14 297
	Min	6 April 2010
	Max	31 March 2016
Hospital length of stay (days)*	N	14 297
	Mean (SD)	33.0 (27.3)
	Median (min, max)	28 (0, 410)
Discharged alive?	N	14 275
	No, n (%)	2677 (18.8)
	Yes, n (%)	11 598 (81.2)

*Length of stay is from admission to discharge alive or until in hospital death. Although we excluded patients discharged alive with a length of stay <3 days, a length of stay <3 days is possible if the patient died within the first 3 days of the IE hospital admission.

IE, infective endocarditis.

### Case-crossover analysis

Case-crossover analysis (table 1) showed that many IPs had no significant IE association. However, our primary step model analysis identified a significant IE association following implantation of cardiac pacemakers/defibrillators (CIEDs) (OR 1.54, 95% CI 1.27 to 1.85, p=<0.001), extractions/surgical tooth removal (OR 2.14, 95% CI 1.22 to 3.76, p=0.047), upper GI (OR 1.58, 95% CI 1.34 to 1.85, p<0.001) and lower GI endoscopic procedures (OR 1.66, 95% CI 1.35 to 2.04, p<0.001) and bone marrow biopsy (OR 1.76, 95% CI 1.16 to 2.69, p=0.039). All except extractions/surgical tooth removal were also significantly associated with IE using the alternative hinge model analysis. Hinge model analysis also demonstrated a significant association with IE following blood transfusion/red cell or plasma exchange (OR 1.2, 95% CI 1.07 to 1.35, p=0.012) and bronchoscopic procedures (OR 1.33, 95% CI 1.06 to 1.68, p=0.049). In the sensitivity analyses, extractions and upper and lower gastrointestinal (GI) endoscopy remained significantly associated with IE when a 4-month (but not a 6 month) case period was used (online supplemental tables S6 and S7). The remaining procedures were neither statistically nor clinically significant, comprising small effects (OR below 1.05) and/or infrequent procedures (<10 per month in control periods).

For IPs with evidence of an association with IE, the absolute risk increase (attributable risk) was small for low/unknown risk and moderate risk patients, with estimated attributable risk below 1 per 100 000 procedures for those at low/unknown IE risk and below 4 per 100 000 for those at moderate IE risk (figure 3). The absolute risk was greatest for those at high IE risk, with the absolute risk being highest for those at high IE risk undergoing dental extractions/surgical removal of teeth (49.5 per 100 000 procedures).

## Discussion

Previous IE guidelines recommended AP before various IPs (online supplemental table S1). These recommendations have been successively abandoned due to lack of evidence to support an association between them and subsequent IE. The exception is AP before IDPs in high-risk individuals, which is still recommended outside the UK.^14 15^ A recent Swedish national study, which will be referred to throughout this discussion, found an association between IE and many IPs previously recommended for AP cover, raising the possibility that withdrawal of AP for these may have been premature.^8^ To explore this, we performed a case-crossover study to determine any temporal association between these IPs and subsequent IE.

### Cardiac procedures

Device infection is a well-recognised complication of CIED implantation, and surveys suggest that most CIED implantations in England were AP covered.^16 17^ Nonetheless, there was considerable variation in the AP regimens used.^16 17^ Concerns about this led to the first UK CIED infection prevention guidelines in 2015.^18^ The incidence of IE following CIED insertion has been calculated at 550 cases/million procedures per year.^19^ Despite it being likely that most CIED implantations were covered by AP, we identified a significantly increased risk of IE in the first 3 months after CIED implantation. The attributable risk was 23.8 per 100 000 procedures for those at high IE risk and 1.8 per 100 000 procedures for those at moderate IE risk. These data suggest that AP cover of CIED implantation was not complete at the time of the study and more may need to be done to improve the effectiveness of CIED infection prevention protocols. It is notable that despite the introduction of UK-wide CIED infection prevention guidelines shortly before the end of this study,^18^ current UK IE prevention guidelines contradict these by recommending against the use of AP to prevent IE and failing to mention the IE risk posed by CIED implantation.^20^

### Dental procedures

During the study period, 294 034 IDPs were performed in English hospitals (of which 70.2% were extractions/surgical tooth removal, 23.8% other surgical procedures and 5.6% scaling procedures). We identified a significant association between extractions/surgical tooth removal and IE. Although no association was identified for other IDPs, the number of these performed in hospital settings was probably too low to detect a significant association. The increase in attributable risk per 100 0000 extractions/surgical tooth removal was 49.5 for those at high risk and 3.9 for those at moderate risk. A large recent study of US dentists also demonstrated a significant association between IDPs (particularly extractions and surgical procedures) and IE that was significantly reduced by AP.^21^ Regrettably, a similar study of dentists in England proved impossible due to inadequacies in data recording. Consistent with the previous observations, most guidelines (except NICE) currently recommend AP in high IE risk patients before IDPs.^9 14 15^

### GI procedures

We identified a statistically significant association between upper (gastric, duodenum, jejunum, ileum) and lower (colon, sigmoid, rectum) GI endoscopic procedures and IE. The increase in attributable risk was, respectively, 25.1 and 28.7 per 100 000 procedures for those at high IE risk undergoing upper or lower GI endoscopy and 2.0 and 2.2 per 100 000 procedures for those at moderate risk. A subanalysis identified no significant difference in the association between upper and lower GI endoscopy and subsequent IE between endoscopy procedures that involved an intervention, for example, a biopsy, and those that did not.

AP was previously recommended before GI endoscopy procedures, and they were also significantly associated with IE in the Swedish study.^8^

Two case series have identified IE following endoscopy,^22 23^ and elevated IE incidence has been noted in elderly high IE risk patients following colonoscopy.^24^ Nonetheless, current IE prevention guidelines do not recommend AP in these settings. One explanation for an association between colonoscopy and IE is that *Streptococcus gallolyticus* IE is associated with colorectal cancer in the elderly or immunocompromised. Indeed, clinicians are advised to exclude colorectal cancer in patients with *Streptococcus gallolyticus* bacteraemia.^25^ However, this does not explain the strong association between upper GI endoscopy and IE and could only explain a small proportion of lower GI endoscopy-associated IE.

We found no association between endoscopic retrograde cholangio-pancreatic (ERCP) and IE. This could be because ERCP patients are frequently already receiving antibiotics for cholangitis or because AP to prevent local infection is recommended in several situations for UK patients undergoing ERCP.^26^

### Haematology procedures

There was a significant association in both the primary step and alternative hinge-model analyses between bone marrow biopsy and IE. There was also an association between blood transfusion, red cell or plasma exchange and IE in the hinge analysis. Neither procedure has previously been recommended for AP cover (online supplemental table S1). We included them because the Swedish study found both significantly associated with IE (RR 4.67, 95% CI 1.34 to 16.24, and RR 6.69, 95% CI 4.43 to 10.11, respectively).^8^

Although these associations may be valid, they could also be explained by diagnostic bone marrow biopsy or therapeutic transfusions, particularly if haematological malignancy is suspected in the weeks before an IE diagnosis is confirmed. This is not uncommon since IE may present with features similar to haematological malignancy. Further investigation into this possible association is essential before drawing any conclusions.

### Respiratory procedures

Most early guidelines recommended AP before bronchoscopy, and our alternative hinge analysis and the Swedish study^8^ identified an association between bronchoscopy and subsequent IE. Furthermore, our attributable risk estimate was 38 additional IE cases per 100 000 procedures for those at high IE risk. Bacteraemia is a recognised complication of bronchoscopy.^27^ Nonetheless, consistent with NICE guidance,^20^ current British Thoracic Society guidelines recommend against the use of AP to prevent IE in those undergoing flexible bronchoscopy.^28^

### Other procedures

We detected no association between ENT, skin or obstetrics and gynaecology procedures and IE. Indeed, the number of these procedures was extremely low over the 15 months before IE admission.

Although cystoscopy and endoscopic prostate procedures were previously recommended for AP cover, and the Swedish study found a significant association between these procedures and IE,^8^ we found no significant association. Antibiotic use to prevent postprocedural urinary tract infections is common and could have masked any relationship in our study. Indeed, another UK study identified a significant association between urological procedures and IE,^29^ so further investigation is warranted.

### Sensitivity analysis

Sensitivity analysis showed the association between extractions, upper or lower GI endoscopy and subsequent IE was sustained for 4 (but not 6) months. The hinge model analysis confirmed the associations identified with the primary step model analysis but identified two more (bronchoscopy and transfusion/red cell/plasma exchange).

The Swedish study did not investigate IDPs, but most IPs we identified as significantly associated with IE were also identified in the Swedish study.^8^ We could not, however, confirm all associations identified in the Swedish study, and the relative risk values they identified were higher than the comparable ORs we found. The reasons for this are: first, the Swedish study screened all inpatient and outpatient IPs to identify associations with IE; we only studied those previously recommended for AP or identified with a positive association in the Swedish study. Second, although both studies used a case-crossover methodology and a 3-month case period, different control periods were used. The Swedish study used a 3-month control period, 1 year before the case period, while we used the preceding 12 months. Sampling the control frequency over an entire year is twice as efficient as sampling equal duration case and control periods.^10^ Finally, with increasing numbers of IPs being performed, using a 1-year control period, and adjusting the ORs to take account of the date of each procedure, allowed us to correct for trends in procedure numbers. This means our adjusted ORs are often smaller but may better reflect any actual association between these IP and IE.

### Study limitations

Misclassification is possible in administrative databases, particularly for challenging diagnoses such as IE. Nonetheless, a recent analysis of English IE admissions showed that the IE definition we used had the best overall balance between sensitivity, positive predictive value (PPV) and negative predictive value (NPV) for identifying modified Duke criteria positive IE cases (sensitivity 0.65, specificity 0.91, PPV 0.80, NPV 0.82).^7^ Administrative databases also afford larger sample sizes, and this study captured the entire spectrum of IE-related hospitalisations in England, removing the potential for referral bias. Furthermore, case-crossover analysis with constant intrasubject characteristics (eg, age, sex, comorbidities), and each individual serving as their control, eliminated control selection bias and confounding.^30^

Although our study recorded all IPs performed, this was not the case for IDPs. Most dental procedures are performed in general dental practice and only a minority in hospitals. This may explain our failure to detect an association between some IDPs and IE and could underestimate any association. Nonetheless, we demonstrated a significant association between extractions (including surgical tooth removal) and subsequent IE.

Our study used ICD-10 and OPCS-4 codes to stratify IE cases into those previously at high, moderate or low/unknown IE risk. However, records of predisposing procedures or conditions were incomplete before January 2000, resulting in the potential misclassification of some high-risk or moderate-risk individuals as low/unknown risk.

Although we wanted to provide details of the causal organisms for IE cases, this proved impossible since there is no requirement to record secondary or supplemental ICD-10 codes on which causal organism data depend and were missing in many cases. This, and the lack of specific ICD-10 codes for oral viridans group streptococci, enterococci and other organisms, made the accurate evaluation of IE microbiology impossible.

## Conclusions

We report a significant association between implantation of CIEDs, upper and lower GI endoscopy, bronchoscopy, and dental extractions (including surgical tooth removal), and subsequent IE. These procedures resulted in an additional 14.3–49.5 IE cases/100 000 procedures in those at high IE risk and an additional 1.1–3.9 IE cases/100 000 procedures in those at moderate risk. These data support a reconsideration of the possible role of preprocedural AP for these procedures in those at high IE risk.

## Data Availability

No data are available. The original data (from which the aggregated data shown in this report are derived) are the subject of data sharing agreements between the University of Sheffield and NHS Digital. These agreements restrict data sharing and require its destruction after study completion. We are therefore unable to share the original data, but they may be obtained by application to NHS Digital (https://digital.nhs.uk) after appropriate regulatory approval.
