# Supplementary material for: Temporal association between invasive procedures and infective endocarditis
Source: Heart. 2022 Sep 22;109(3):223–31. doi: 10.1136/heartjnl-2022-321519 (PMC9872236; doi:10.1136/heartjnl-2022-321519)
Supplement: Supplementary data [file heartjnl-2022-321519supp001.pdf]

# Supplementary Appendix

# Temporal Association Between Invasive Procedures and Infective Endocarditis

## Brief Title – Endocarditis and invasive procedures

**Martin H. Thornhill, MBBS, BDS, PhD[a,b], Annabel Crum, BSc[c], Richard Campbell, MPH[c], Ellen C. Lee BSC, [M.Scc], Veronica Fibisan PhD[c], Mark J. Dayer, MBBS, PhD[d], Bernard D. Prendergast, BM, BS, DM[e], Peter B. Lockhart, DDS[b], Larry M. Baddour, MD[g], Jon Nicholl, DSc[c].**

[a]Unit of Oral & Maxillofacial Medicine Surgery and Pathology, School of Clinical Dentistry, University of Sheffield, Sheffield, UK; [c]School of Health and Related Research, University of Sheffield, Sheffield, UK; [b]Department of Oral Medicine, Carolinas Medical Center – Atrium Health, Charlotte, NC; [c]IBM Watson Health, Ann Arbor, MI; [d]Department of Cardiology, Somerset Foundation Trust, Taunton, Somerset, UK; [e]Department of Cardiology, St Thomas' Hospital, London, UK; [f]Cardiovascular Medicine Division, Brigham and Women's Hospital, and Harvard Medical School, Boston, MA; [g]Division of Infectious Diseases, Mayo Clinic College of Medicine and Science, Rochester, MN.

**Correspondence to:**

Prof. Martin Thornhill, Unit of Oral & Maxillofacial Medicine Surgery and Pathology, University of Sheffield School of Clinical Dentistry, Claremont Crescent, Sheffield S10 2TA, UK. Tel: +44 (0)751-555-2925, Email: m.thornhill@sheffield.ac.uk



Thornhill MH, *et al. Heart* 2023; 109:223–231. doi: 10.1136/heartjnl-2022-321519

# Contents

**Page**

**Abbreviations.** **3**

**Supplementary Methods** **4**

**Table S1.** **6**
**Invasive procedures identified for antibiotic prophylaxis by previous guidelines or with positive association with subsequent infective endocarditis.**

**Table S2.** **7**
**Codes used to identify those at high-risk of IE. Primary and secondary codes used.**

**Table S3.** **10**
**Codes used to identify those at moderate-risk of IE.**

**Table S4.** **12**
**Summary of Invasive Procedure (IPs) OPCS-4 Codes.**

**Table S5.** **14**
**Individual OPCS-4 Invasive Procedure (IPs) Code Descriptions.**

**Table S6.** **34**
**Case-crossover step-analysis comparing the incidence of invasive procedures (IPs) using a 4-month case period and preceding 11-month control period for 17,732 patients admitted with IE.**

**Table S7.** **36**
**Case-crossover step-analysis comparing the incidence of invasive procedures (IPs) using a 6-month case period and preceding 9-month control period for 17,732 patients admitted with IE.**

**Figure S1.** **38**
**Incidence of different invasive procedures (IPs) over the 15 months before infective endocarditis (IE) hospital admission.**

**References.** **40**

## Abbreviations:

AHA = American Heart Association
AP = Antibiotic prophylaxis
BCS = British Cardiac Society
BSAC = British Society for Antimicrobial Chemotherapy
CABG = Coronary artery bypass graft
CI = Confidence interval
ENT = Ear nose and throat
ESC = European Society for Cardiology
ERCP = Endoscopic retrograde cholangio-pancreatic procedures
IDPs = Invasive-dental procedures
IPs = Invasive procedures
IE = Infective endocarditis
NICE = National Institute for Health and Care Excellence
OR = Odds ratio
RR = Relative risk
UK = United Kingdom
US = United States of America

## Supplementary Methods

Analyses:

Case-Crossover studies compare exposure to possible trigger events (in this case the incidence of invasive procedures [IP]) for an outcome (in this case admission for infective endocarditis [IE]) during an exposure window (in this case the 3-month case-period before hospital admission for infective endocarditis [IE]) when exposure may lead to the outcome and compares this with the exposure in an earlier control-period (in this case the incidence of IP in the 12 months preceding the case-period). In its simplest form this involves comparing the monthly incidence of the IP in the case-period with the incidence in the control period. If a causal relationship exists between the IP and the outcome IE, then one would expect the mean IP incidence in the case-period to exceed the mean IP incidence in the control-period i.e., there would be a step increase in the IP incidence in the control period on transitioning into the case period. Hence the term "step-model". This is depicted in the figure below by the graph labelled 'Sep-Model'. The step increase between the control and case period means can be seen. This is what we describe as the unadjusted "step-model".

*Unadjusted step-model analysis:*

For each procedure, the binary outcome (procedure occurred, yes/no) was modelled longitudinally. Whilst it is possible to have multiple procedures within the same month, the vast majority of procedures considered here occur only once if at all. Furthermore, multiple procedures within the same month are likely to relate to one episode for these procedures. For both of these reasons, (simplicity and avoidance of double counting) we chose to limit analysis to yes/no incidence. The unadjusted model considers the probability of an invasive procedure during a month within the "case period" (3 months prior to IE), compared to the probability of an invasive procedure in the remaining months of the study (4-15 prior to IE). This is referred to as a "step-model" as the proportion of patients undergoing a procedure is assumed to follow a step-change once the patient enters the case period:

$$logit(p_{it}) = \alpha + \beta_{case} \times I_{(t)} + \gamma_i$$

Where $p_{it}$ denotes the probability that the $i^{th}$ patient recorded a procedure in time-period $t$ (1 to 15), $I_{(t)}$ is equal to 1 if the current timepoint is within 3 months of the IE and 0 otherwise, $\beta_{case}$ is the coefficient (log-odds) for the procedure in the case period compared to the non-case period, $\gamma_i$ is a Normally distributed random effect $\gamma_i$ specific to the patient. This analysis compares monthly occurrence during "case" periods to "control" periods but is biased (exaggerated) if the incidence of procedures increases with time.

To address this, we defined an adjusted 'step model' as our primary outcome. This corrected for the background increase in IP incidence and is represented in the figure below by the graph labelled 'Adjusted Step-Model'. Despite adjusting for the background increase in IP incidence, there is still a step increase in the incidence between the case- and control-periods caused by any association between the IP and subsequent IE (hence the term adjusted "step-model"). The important thing to note is that the unadjusted "step-model" (that represents most case-crossover studies on this subject e.g., the Janszky study (Janszky et al, J. Am. Coll.

Cardiol. (2018) 71(24)2744-2752)) will tend to exaggerate or overestimate the Odds or RR in a situation where the incidence of the potential trigger (IP in this case) is increasing over time. Whereas the adjusted "step-model' (our primary outcome) helps to correct for this but results in a lower OR/RR, – as discussed in the last two sentences of the Discussion section on 'Sensitivity Analysis'.

*Adjusted step-model analysis:*

The adjusted model builds on the unadjusted model which also incorporates the association between procedure and calendar time:

$$logit(p_{it}) = \alpha + \beta_{case} \times I_{(t)} + \beta_{time} \times d_t + \gamma_i$$

Where $d_t$ is the calendar date (1st April 2010 to 31st March 2016), $\beta_{time}$ is the coefficient (log-odds) for the temporal association, and $p_{it}$ , $I_{(t)}$ and $\gamma_i$ are as above. This analysis models associations both temporally and with procedure but models the change as a jump at three months prior to IE and was chosen as our primary analysis.

An alternative approach is to consider the slope of the IP incidence in the case- and control-periods as continuous (i.e., without a step change) but with a change of slope (or trajectory) occurring at the point of transition from the control- to the case-period (i.e., with a hinge point in the slope). This is depicted in the figure below by the graph labelled 'Hinge-model'.

*Hinge-model analysis:*

The hinge-model analysis allows a gradual separation of the curves during the case period, as opposed to the step (or "jump") the previous two models. Like the adjusted step-model, this also incorporates the association between procedure and calendar time:

$$logit(p_{it}) = \alpha + \beta_{case} \times I_{(t)} \times t^* + \beta_{time} \times d_t + \gamma_i$$

Where $t^*$ equal to the study month. This analysis is more biologically plausible and most sensitive to detecting an association where one exists but cannot directly estimate the attributable risk. Since we wanted to estimate this, and since the incidence of many procedures appeared to increase with time, the adjusted step-model analysis was selected as our primary analysis.

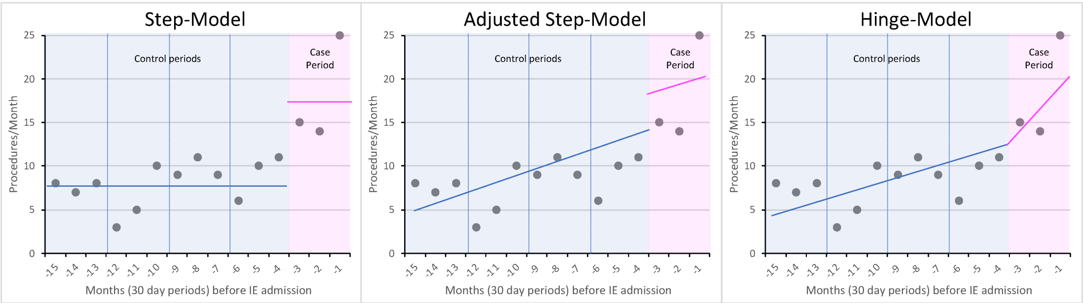

## Table S1. Invasive procedures identified for antibiotic prophylaxis by previous guidelines or with positive association with subsequent infective endocarditis

| Invasive Procedures (IPs) | BCS 2004[1] | ESC 2004[2] | AHA 1997[3] | Janszky et al. 2018[4] Inpatient IPs RR (95% CI) |
|---|---|---|---|---|
| **GI Procedures** | | | | |
| Upper GI Endoscopy with/without biopsy | ✓ | - | ✓† | 3.97 (2.68-5.88) |
| Lower GI Endoscopy with/without biopsy | ✓ | - | ✓† | 2.82 (1.42-5.61) |
| ERCP (Endoscopic Retrograde Cholangio-Pancreatography) | ✓ | ✓ | ✓ | 3.60 (1.34-9.70) |
| Colonic Surgery | ✓ | - | ✓ | - |
| **GU Procedures** | | | | |
| Endoscopic prostate procedures | ✓ | ✓ | ✓ | - |
| Cystoscopy and endoscopic urological procedures | ✓ | ✓ | ✓ | 4.40 (1.67-11.62) |
| **Obstetric & Gynaecological Procedures** | | | | |
| Caesarean section | ✓ | - | - | - |
| Vaginal delivery | ✓ | ✓§ | ✓† | - |
| Abortion/dilatation and curettage (D&C) | ✓ | ✓§ | - | 3.00 (1.81-4.98) |
| **Respiratory Procedures** | | | | |
| Bronchoscopic procedures (esp. rigid) | ✓ | ✓ | ✓ | 16.00 (2.12-120.65) |
| **Cardiac Procedures** | | | | |
| Implantation of pacemakers/defibrillators | ✓ | - | - | 9.75 (3.48-27.28) |
| Percutaneous valve procedures | ✓ | - | - | - |
| Percutaneous coronary procedures/stents | ✓ | - | - | 3.50 (1.41-8.67) |
| Coronary artery bypass graft (CABG) | - | - | - | 13.8 (5.57-34.21) |
| Coronary angiography | | - | - | 4.23 (2.93-6.11) |
| **ENT Procedures** | | | | |
| Tonsillectomy/adenoidectomy | ✓ | ✓ | ✓ | 2.33 (0.60-9.02) |
| Nasal packing/nasal intubation | ✓ | - | - | - |
| **Dermatological Procedures** | | | | |
| Skin suturing, drainage or wound management | ✓ | - | - | 7.00 (0.86-56.89) |
| **Haematological Procedures** | | | | |
| Blood transfusion/red cell/plasma exchange | - | - | - | 6.69 (4.43-10.11) |
| Bone marrow puncture | - | - | - | 4.67 (1.34-16.24) |
| **Dental Procedures** | | | | |
| Dental extractions | ✓ | ✓ | ✓ | - |
| Other oral surgical procedures | ✓ | ✓ | ✓ | - |
| Scaling of teeth | ✓ | ✓ | ✓ | - |
| Endodontic treatment | ✓ | ✓ | ✓ | - |

Notes: This table shows those invasive procedures (IPs) for which antibiotic prophylaxis (AP) was recommended for those at moderate- or high-risk of infective endocarditis (IE) (i) by the 2004 British Cardiac Society (BCS) guidance,[1] (ii) by the 2004 European Society for Cardiology (ESC) guidance,[2] or (iii) the 1997 American Heart Association (AHA) guidelines.[3] It also shows the increased relative risk (RR), with 95% Confidence Intervals, of developing IE after different types of IP that was identified in the 2018 analysis of Swedish national data for hospital admissions between January 1998 and December 2011 by Janszky et al.[4] The 2009 ESC guidelines[5] and the 2007 AHA guidelines[6] recommended against the use of AP for all IPs in those at moderate-IE-risk and for all IPs, except dental IPs, in those at high-IE-risk. The 2008 NICE guidelines in the UK recommended the complete cessation of AP for all IPs, including dental procedures.[7] TOE= Transoesophageal echocardiography, ✓ = antibiotic prophylaxis recommended, ✓† = prophylaxis recommended as optional for high-risk patients, ✓§ = antibiotic prophylaxis recommended in the presence of infection.

**Table S2.**

**Codes used to identify those at high-risk of IE. Primary and secondary codes used (except I38X for which primary diagnosis code only was used).**

| Cardiac Condition | ICD-10 Diagnosis Codes and OPCS-4 Procedure codes For Identifying Those at High-Risk of IE[1] |
|---|---|
| Previous IE | ICD-10 Diagnosis Codes:<br>I330 Acute and subacute infective endocarditis<br>I339 Acute endocarditis, unspecified<br>I38X Endocarditis, valve unspecified<br>I390 Endocarditis and mitral valve disorders in disease classified elsewhere<br>I391 Endocarditis and aortic valve disorders in disease classified elsewhere<br>I392 Endocarditis and tricuspid valve disorders in disease classified elsewhere<br>I393 Endocarditis and pulmonary valve disorders in disease classified elsewhere<br>I394 Endocarditis and multiple valve disorders in disease classified elsewhere<br>I398 Endocarditis, valve unspecified in disease classified elsewhere<br>B376 Candidal endocarditis<br>T826 Infection and inflammatory reaction due to cardiac valve prosthesis |
| Prosthetic replacement of heart valve | OPCS-4 Procedure Codes:<br>K251 Allograft replacement of mitral valve<br>K252 Xenograft replacement of mitral valve<br>K253 Prosthetic replacement of mitral valve<br>K254 Replacement of mitral valve NEC<br>K261 Allograft replacement of aortic valve<br>K262 Xenograft replacement of aortic valve<br>K263 Prosthetic replacement of aortic valve<br>K264 Replacement of aortic valve NEC<br>K271 Allograft replacement of mitral valve<br>K272 Xenograft replacement of mitral valve<br>K273 Prosthetic replacement of mitral valve<br>K274 Replacement of mitral valve NEC<br>K281 Allograft replacement of pulmonary valve<br>K282 Xenograft replacement of pulmonary valve<br>K283 Prosthetic replacement of pulmonary valve<br>K284 Replacement of pulmonary valve NEC<br>K291 Allograft replacement of valve of heart NEC<br>K292 Xenograft replacement of valve of heart NEC<br>K293 Prosthetic replacement of valve of heart NEC<br>K294 Replacement of valve of heart NEC<br>K297 Replacement of truncal valve<br>K331 Aortic root replacement using pulmonary valve autograft with right ventricle to pulmonary artery valved conduit<br>K332 Aortic root replacement using pulmonary valve autograft with right ventricle to pulmonary artery valved conduit and aortoventriculoplasty<br>K333 Aortic root replacement using homograft<br>K334 Aortic root replacement using mechanical prosthesis<br>K336 Aortoventriculoplasty with pulmonary valve autograft<br>K357 Percutaneous transluminal pulmonary valve replacement |
| Valve repair using prosthetic material | OPCS-4 Procedure Codes:<br>K255 Mitral valve repair NEC<br>K258 Other specified plastic repair of mitral valve<br>K259 Unspecified plastic repair of mitral valve<br>K265 Aortic valve repair NEC<br>K268 Other specified plastic repair of aortic valve<br>K269 Unspecified plastic repair of aortic valve<br>K275 Repositioning of tricuspid valve |

| | |
|---|---|
| | K276 Tricuspid valve repair NEC<br>K278 Other specified plastic repair of tricuspid valve<br>K279 Unspecified plastic repair of tricuspid valve<br>K285 Pulmonary valve repair NEC<br>K288 Other specified plastic repair of pulmonary valve<br>K289 Unspecified plastic repair of pulmonary valve<br>K295 Repair of valve of heart NEC<br>K296 Truncal valve repair<br>K298 Other specified plastic repair of unspecified valve of heart<br>K299 Unspecified plastic repair of unspecified valve of heart<br>K301 Revision of plastic repair of mitral valve<br>K302 Revision of plastic repair of aortic valve<br>K303 Revision of plastic repair of tricuspid valve<br>K304 Revision of plastic repair of pulmonary valve<br>K305 Revision of plastic repair of truncal valve<br>K308 Other specified revision of plastic repair of valve of heart<br>K309 Unspecified revision of plastic repair of valve of heart<br>K335 Aortic root replacement NEC<br>K341 Annuloplasty of mitral valve<br>K342 Annuloplasty of tricuspid valve<br>K343 Annuloplasty of valve of heart NEC<br>K358 Other specified therapeutic transluminal operations on valve of heart<br>K359 Unspecified therapeutic transluminal operations on valve of heart |
| Prosthetic heart or ventricular assist device | OPCS-4 Procedure Codes:<br>K023 Implantation of prosthetic heart<br>K025 Revision of implantation of prosthetic heart<br>K541 Open implantation of ventricular assist device |
| Congenital Heart Condition (CHC) in whom a palliative shunt or conduit has been used | OPCS-4 Procedure Codes:<br>K041 Repair of tetralogy of Fallot using valved right ventricular outflow conduit<br>K042 Repair of tetralogy of Fallot using right ventricular outflow conduit NEC<br>K063 Left ventricle to aorta tunnel with right ventricle to pulmonary artery valved conduit<br>K171 Total cavopulmonary connection with extracardiac inferior caval vein to pulmonary artery conduit<br>K173 Aortopulmonary reconstruction with systemic to pulmonary arterial shunt<br>K174 Aortopulmonary reconstruction with right ventricle to pulmonary arterial valveless conduit<br>K181 Creation of valved conduit between atrium and ventricle of heart<br>K182 Creation of valved conduit between right atrium and pulmonary artery<br>K183 Creation of valved conduit between right ventricle of heart and pulmonary artery<br>K184 Creation of valved conduit between left ventricle of heart and aorta<br>K185 Revision of valved cardiac conduit<br>K186 Creation of valved conduit between left ventricle of heart pulmonary artery<br>K187 Replacement of valved cardiac conduit<br>K188 Other specified creation of valved cardiac conduit<br>K189 Unspecified creation of valved cardiac conduit<br>K191 Creation of conduit between atrium and ventricle of heart NEC<br>K192 Creation of conduit between right atrium and pulmonary artery NEC<br>K193 Creation of conduit between right ventricle of heart and pulmonary artery NEC<br>K194 Creation of conduit between right ventricle of heart and vena cava<br>K195 Creation of conduit between left ventricle of heart and aorta NEC<br>K196 Revision of cardiac conduit NEC<br>K198 Other specified creation of other cardiac conduit<br>K199 Unspecified creation of other cardiac conduit<br>K761 Percutaneous transluminal balloon dilatation of cardiac conduit<br>K768 Other specified transluminal operations on cardiac conduit<br>K769 Unspecified transluminal operations on cardiac conduit<br>L051 Creation of shunt to main pulmonary artery from ascending aorta using interposition tube prosthesis |

| | |
|---|---|
| | L052 Creation of shunt to right pulmonary artery from ascending aorta using interposition tube prosthesis<br>L053 Creation of shunt to left pulmonary artery from ascending aorta using interposition tube prosthesis<br>L054 Percutaneous transluminal balloon dilatation of interposition tube prosthesis between pulmonary artery and aorta<br>L058 Other specified creation of shunt to pulmonary artery from aorta using interposition tube prosthesis<br>L059 Unspecified creation of shunt to pulmonary artery from aorta using interposition tube prosthesis<br>L071 Creation of shunt to right pulmonary artery from right subclavian artery using interposition tube prosthesis<br>L072 Creation of shunt to left pulmonary artery from left subclavian artery using interposition tube prosthesis<br>L074 Percutaneous transluminal balloon dilatation of interposition tube prosthesis between pulmonary artery and subclavian artery<br>L078 Other specified creation of shunt to pulmonary artery from subclavian artery using interposition tube prosthesis<br>L079 Unspecified creation of shunt to pulmonary artery from subclavian artery using interposition tube prosthesis |
| Unrepaired cyanotic congenital heart condition (CHC)[2] | <u>ICD-10 Diagnosis Codes:</u><br>Q200 Common arterial trunk<br>Q201 Double outlet right ventricle<br>Q202 Double outlet left ventricle<br>Q203 Discordant ventriculoarterial connection<br>Q204 Double inlet ventricle<br>Q205 Discordant atrioventricular connection<br>Q212 Atrioventricular septal defect<br>Q213 Tetralogy of Fallot<br>Q214 Aortopulmonary septal defect<br>Q262 Total anomalous pulmonary venous connection |
| Completely repaired CHC defect with prosthetic material or device, whether placed by surgery or catheter intervention, during first 6 months after the procedure only.[3] | <u>OPCS-4 Procedure Codes:</u><br>K091 Repair of defect of atrioventricular septum using dual prosthetic patches<br>K092 Repair of defect of atrioventricular septum using prosthetic patch NEC<br>K101 Repair of defect of interatrial septum using prosthetic patch<br>K111 Repair of defect of interventricular septum using prosthetic patch<br>K117 Repair of defect of inter ventricular septal defect using intraoperative transluminal prosthesis<br>K121 Repair of defect of septum of heart using prosthetic patch NEC<br>K131 Percutaneous transluminal repair of defect of interventricular septum using prosthesis<br>K132 Percutaneous transluminal repair of defect of interventricular septum NEC<br>K133 Percutaneous transluminal repair of defect of interatrial septum using prosthesis<br>K134 Percutaneous transluminal repair of defect of interatrial septum NEC<br>K135 Percutaneous transluminal repair of defect of unspecified septum using prosthesis<br>K138 Other specified transluminal repair of defect of interatrial septum<br>K139 Unspecified transluminal repair of defect of interatrial septum<br>K163 Percutaneous transluminal atrial septum fenestration closure with prosthesis<br>K165 Percutaneous transluminal closure of patent oval foramen with prosthesis<br>L031 Percutaneous transluminal prosthetic occlusion of patent ductus arteriosus<br>L101 Repair of pulmonary artery using prosthesis<br>L233 Plastic repair of aorta using patch graft |

## Table S3.

## Codes used to identify those at moderate-risk of IE

| Cardiac Condition | ICD-10 Diagnosis Codes and OPCS-4 Procedure codes For Identifying Those at High-Risk of IE[1] |
|---|---|
| Previous Rheumatic Fever | **ICD-10 Diagnosis Codes:**<br>I011 Acute rheumatic endocarditis<br>I018 Other acute rheumatic heart disease<br>I019 Acute rheumatic heart disease, unspecified<br>I020 Rheumatic chorea with heart involvement<br>I050 Rheumatic mitral stenosis<br>I051 Rheumatic mitral insufficiency<br>I052 Rheumatic mitral stenosis with insufficiency<br>I058 Other mitral valve disease<br>I059 Rheumatic mitral valve disease, unspecified<br>I060 Rheumatic aortic stenosis<br>I061 Rheumatic aortic insufficiency<br>I062 Rheumatic aortic stenosis with insufficiency<br>I068 Other rheumatic aortic valve disease<br>I069 Rheumatic aortic valve disease, unspecified<br>I070 Rheumatic tricuspid stenosis<br>I071 Rheumatic tricuspid insufficiency<br>I072 Rheumatic tricuspid stenosis with insufficiency<br>I078 Other rheumatic tricuspid valve disease<br>I079 Rheumatic tricuspid valve disease, unspecified<br>I080 Disorders of both mitral and aortic valves<br>I081 Disorders of both mitral and tricuspid valves<br>I082 Disorders of both aortic and tricuspid valves<br>I083 Combined disorders of mitral, aortic and tricuspid valves<br>I088 Other multiple valve disease<br>I089 Multiple valve diseases, unspecified<br>I090 Rheumatic myocarditis<br>I091 Rheumatic disease of the endocardium, valve unspecified<br>I098 Other specified rheumatic heart disease<br>I099 Rheumatic heart disease, unspecified |
| Non-Rheumatic Valve Disease | **ICD-10 Diagnosis Codes:**<br>I340 Mitral valve insufficiency<br>I341 Mitral valve prolapse<br>I342 Nonrheumatic mitral valve stenosis<br>I348 Other nonrheumatic mitral valve disorders<br>I349 Nonrheumatic mitral valve disorder, unspecified<br>I350 Aortic valve stenosis<br>I351 Aortic valve insufficiency<br>I352 Aortic valve stenosis with insufficiency<br>I358 Other nonrheumatic aortic valve disorders<br>I359 Nonrheumatic aortic valve disorder, unspecified<br>I360 Tricuspid valve stenosis<br>I361 Tricuspid valve insufficiency<br>I362 Tricuspid valve stenosis with insufficiency<br>I368 Other nonrheumatic tricuspid valve disorders<br>I369 Nonrheumatic tricuspid valve disorder, unspecified<br>I370 Pulmonary valve stenosis<br>I371 Pulmonary valve insufficiency<br>I372 Pulmonary valve stenosis with insufficiency<br>I378 Other nonrheumatic pulmonary valve disorders<br>I379 Nonrheumatic pulmonary valve disorder, unspecified |



Thornhill MH, *et al. Heart* 2023; 109:223–231. doi: 10.1136/heartjnl-2022-321519

| | |
|---|---|
| Hypertrophic cardiomyopathy | **ICD-10 Diagnosis Codes:**<br>I421 Obstructive hypertrophic cardiomyopathy<br>I422 Other hypertrophic cardiomyopathy |
| Congenital valve anomalies | **ICD-10 Diagnosis Codes:**<br>Q221 Congenital pulmonary valve stenosis<br>Q222 Congenital pulmonary valve insufficiency<br>Q223 Other congenital malformations of pulmonary valve<br>Q224 Congenital tricuspid valve stenosis<br>Q225 Ebstein anomaly<br>Q228 Other congenital malformations of tricuspid valve<br>Q229 Congenital malformations of tricuspid valve, unspecified<br>Q230 Congenital stenosis of aortic valve<br>Q231 Congenital insufficiency of aortic valve<br>Q232 Congenital mitral valve stenosis<br>Q233 Congenital mitral valve insufficiency<br>Q238 Other congenital malformations of aortic and mitral valves<br>Q239 Congenital malformations of aortic and mitral valves, unspecified |

## Table S4

## Summary of Invasive Procedure (IPs) OPCS-4 Codes

| Procedures | Paper (RR)* | OPCS-4 Codes | Most Likely Organisms |
|---|---|---|---|
| **Haematology Procedures** | | | |
| Blood transfusion/red cell or plasma exchange | 7 | X32.1-X34.9 (3 Character – X32–X34) | Staph |
| Bone marrow puncture | 16 | W36.5, Y66.7<br>(Must use 4 character codes) | Staph |
| | | | |
| **GI Procedures** | | | |
| Oesophageal endoscopic procedures (all) | | G14.1-G20.9<br>U20.2<br>(3 Character – G14–G20 + U20.2) | |
| - Transoesophageal echocardiography (TOE) | | U20.2 | |
| - Oesophageal endoscopic procedures (excluding TOE) | | G14.1-G20.9<br>(3 Character – G14–G20) | |
| Upper GI endoscopic procedures (gastric, jejunum, ileum) | 4 | G42.1-G46.9<br>G54.1-G55.9<br>G64.1-G65.9<br>G79.1-G79.9<br>G80.1-G80.9<br>(3 Character – G42-G46, G54-G55, G64-G65 + G79-G80) | Enterococci |
| Lower GI endoscopic procedures (including sigmoid and rectum) | 3 | H20.1-28.9<br>(3 Character – H20–H28) | Enterococci |
| Colonic surgery (incl appendix) | | H01.1-19.9<br>H29.1-29.9<br>(3 Character – H01-03 (Appendix), H04–H19 and H29 (Colonic surgery)) | |
| Endoscopic Retrograde Cholangio-pancreatic Procedures (ERCP) | | J40-1 - J45.9<br>(3 Character – J40 - J45) | Enterococci |
| | | | |
| **GU Procedures** | | | |
| Cystoscopic procedures | 4 | M09.1-M11.9<br>M27.1-M30.9<br>M42.1-M45.9<br>(3 Character – M09–M11, M27–M30, M42-M45) | Enterococci |
| Endoscopic prostate procedures | | M65.1-M68.9<br>M70.1-M71.9<br>(3 Character – M65-M68 and M70-M71) | |
| | | | |
| **Respiratory Procedures** | | | |
| Bronchoscopic procedures | | E48.1-51.9 | ? |



Thornhill MH, *et al. Heart* 2023; 109:223–231. doi: 10.1136/heartjnl-2022-321519

| | | | |
|---|---|---|---|
| | | (3 Character – E48-E51) | |
| | | | |
| **ENT procedures** | | | ? |
| Tonsillectomy & Adenoidectomy | 2 | E20.1–E20.9<br>F34.1-34.9, F36.1-F36.9<br>(3 Character – E20, F34, F36) | ? |
| | | | |
| Nasal packing/nasal intubation | | E06.1-E06.9 + X56.1<br>(3 Character – E06 + 4 char X56.1) | |
| | | | |
| **Obstetric & gynae procedures** | | | ? |
| Abortion/dilatation & curettage (D&C) | 3 | Q10.1-11.9<br>(3 Character – Q10, Q11) | |
| Vaginal delivery | | R19.1-24.9<br>(3 Character – R19-R24) | |
| Caesarean delivery | | R17.1-18.9<br>(3 Character – R17, R18) | |
| | | | |
| **Cardiac Procedures** | | | |
| Coronary angiography | 4 | K63.1-63.9, K65.1-K65.9. (3 Character – K63, K65) | Staph |
| Coronary artery bypass | 14 | K40.1-46.9 (3 Character – K40-K46) | Staph |
| Percutaneous coronary procedures and stents | | K49.1-K51.9, K75.1-75.9 (3 Character – K49-51 & K75) | Staph |
| Implantation of cardiac pacemakers/defibrillators | | K59.1-K61.9, K73.1-K73.9 (3 Character – K59-61 & K73) | |
| Percutaneous valve procedures/heart catheterisation | | K35.1-K35.9 (3 Character – K35) | |
| | | | |
| Skin and wound management procedures | 7 | S41.1-S42.9, S47.1-S47.9, S54.1-S57.9 (3 Character – S41-42, S47 & S54-57) | Staph |
| **Dental Procedures** | N/A | | |
| Extractions & surgical removal of teeth | | F09.1-F10.9 (3 Character F09-F10) | OVGS |
| Other oral surgical procedures | | 3 Character F01, F02, F03, F04, F05, F06, F08, F11, F18, F22, F23, F24, F26, F28, F29, F30, F32, F38, F39, F40 and 4 Character F42.1-42.3 | OVGS |
| Endodontic procedures | | F12.1-F12.9 (3 Character F12) | OVGS |
| Scaling and gingival procedures | | F16.4, F20.1-F20.9 (3 Character F20 + 4 Character F16.4) | OVGS |
| Restorative Dental Procedures | | F13.1-F13.5, F13.8-F13.9 F17.1, F17.6 (Use 4-character codes) | |

* Increased relative risk of developing IE within 3 months of this procedure being performed according to the data published by Janszky et al. 2018[4]

## Table S5
## Individual OPCS-4 Invasive Procedure (IPs) Code Descriptions

| Procedure | Code | OPCS-4 Code Description |
|---|---|---|
| **Cardiac Procedures** | | |
| Coronary angiography | K63 | Contrast radiology of heart |
| | K63.1 | Angiocardiography of combination of right and left side of heart |
| | K63.2 | Angiocardiography of right side of heart NEC |
| | K63.3 | Angiocardiography of left side of heart NEC |
| | K63.4 | Coronary arteriography using two catheters |
| | K63.5 | Coronary arteriography using single catheter |
| | K63.6 | Coronary arteriography NEC |
| | K63.8 | Other specified contrast radiology of heart |
| | K63.9 | Unspecified contrast radiology of heart |
| | K65 | Catheterisation of heart |
| | K65.1 | Catheterisation of combination of right and left side of heart NEC |
| | K65.2 | Catheterisation of right side of heart NEC |
| | K65.3 | Catheterisation of left side of heart NEC |
| | K65.4 | Catheterisation of left side of heart via atrial transeptal puncture |
| | K65.8 | Other specified catheterisation of heart |
| | K65.9 | Unspecified catheterisation of heart |
| Coronary artery bypass | K40 | Saphenous vein graft replacement of coronary artery |
| | K40.1 | Saphenous vein graft replacement of one coronary artery |
| | K40.2 | Saphenous vein graft replacement of two coronary arteries |
| | K40.3 | Saphenous vein graft replacement of three coronary arteries |
| | K40.4 | Saphenous vein graft replacement of four or more coronary arteries |
| | K40.8 | Other specified saphenous vein graft replacement of coronary artery |
| | K40.9 | Unspecified saphenous vein graft replacement of coronary artery |
| | K41 | Other autograft replacement of coronary artery |
| | K41.1 | Autograft replacement of one coronary artery NEC |
| | K41.2 | Autograft replacement of two coronary arteries NEC |
| | K41.3 | Autograft replacement of three coronary arteries NEC |
| | K41.4 | Autograft replacement of four or more coronary arteries NEC |
| | K41.8 | Other specified other autograft replacement of coronary artery |
| | K41.9 | Unspecified other autograft replacement of coronary artery |
| | K42 | Allograft replacement of coronary artery |
| | K42.1 | Allograft replacement of one coronary artery |
| | K42.2 | Allograft replacement of two coronary arteries |
| | K42.3 | Allograft replacement of three coronary arteries |
| | K42.4 | Allograft replacement of four or more coronary arteries |
| | K42.8 | Other specified allograft replacement of coronary artery |
| | K42.9 | Unspecified allograft replacement of coronary artery |
| | K43 | Prosthetic replacement of coronary artery |
| | K43.1 | Prosthetic replacement of one coronary artery |
| | K43.2 | Prosthetic replacement of two coronary arteries |
| | K43.3 | Prosthetic replacement of three coronary arteries |
| | K43.4 | Prosthetic replacement of four or more coronary arteries |
| | K43.8 | Other specified prosthetic replacement of coronary artery |
| | K43.9 | Unspecified prosthetic replacement of coronary artery |
| | K44 | Other replacement of coronary artery |
| | K44.1 | Replacement of coronary arteries using multiple methods |
| | K44.2 | Revision of replacement of coronary artery |
| | K44.8 | Other specified other replacement of coronary artery |
| | K44.9 | Unspecified other replacement of coronary artery |
| | K45 | Connection of thoracic artery to coronary artery |
| | K45.1 | Double anastomosis of mammary arteries to coronary arteries |
| | K45.2 | Double anastomosis of thoracic arteries to coronary arteries NEC |



Thornhill MH, *et al. Heart* 2023; 109:223–231. doi: 10.1136/heartjnl-2022-321519

| | | |
|---|---|---|
| | K45.3 | Anastomosis of mammary artery to left anterior descending coronary artery |
| | K45.4 | Anastomosis of mammary artery to coronary artery NEC |
| | K45.5 | Anastomosis of thoracic artery to coronary artery NEC |
| | K45.6 | Revision of connection of thoracic artery to coronary artery |
| | K45.8 | Other specified connection of thoracic artery to coronary artery |
| | K45.9 | Unspecified connection of thoracic artery to coronary artery |
| | K46 | Other bypass of coronary artery |
| | K46.1 | Double implantation of mammary arteries into heart |
| | K46.2 | Double implantation of thoracic arteries into heart NEC |
| | K46.3 | Implantation of mammary artery into heart NEC |
| | K46.4 | Implantation of thoracic artery into heart NEC |
| | K46.5 | Revision of implantation of thoracic artery into heart |
| | K46.8 | Other specified other bypass of coronary artery |
| | K46.9 | Unspecified other bypass of coronary artery |
| Percutaneous coronary procedures and stents | K49 | Transluminal balloon angioplasty of coronary artery |
| | K49.1 | Percutaneous transluminal balloon angioplasty of one coronary artery |
| | K49.2 | Percutaneous transluminal balloon angioplasty of multiple coronary arteries |
| | K49.3 | Percutaneous transluminal balloon angioplasty of bypass graft of coronary artery |
| | K49.4 | Percutaneous transluminal cutting balloon angioplasty of coronary artery |
| | K49.8 | Other specified transluminal balloon angioplasty of coronary artery |
| | K49.9 | Unspecified transluminal balloon angioplasty of coronary artery |
| | K50 | Other therapeutic transluminal operations on coronary artery |
| | K50.1 | Percutaneous transluminal laser coronary angioplasty |
| | K50.2 | Percutaneous transluminal coronary thrombolysis using streptokinase |
| | K50.3 | Percutaneous transluminal injection of therapeutic substance into coronary artery NEC |
| | K50.4 | Percutaneous transluminal atherectomy of coronary artery |
| | K50.8 | Other specified other therapeutic transluminal operations on coronary artery |
| | K50.9 | Unspecified other therapeutic transluminal operations on coronary artery |
| | K51 | Diagnostic transluminal operations on coronary artery |
| | K51.1 | Percutaneous transluminal angioscopy |
| | K51.2 | Intravascular ultrasound of coronary artery |
| | K51.8 | Other specified diagnostic transluminal operations on coronary artery |
| | K51.9 | Unspecified diagnostic transluminal operations on coronary artery |
| | K75 | Percutaneous transluminal balloon angioplasty and insertion of stent into coronary artery |
| | K75.1 | Percutaneous transluminal balloon angioplasty and insertion of 1-2 drug-eluting stents into coronary artery |
| | K75.2 | Percutaneous transluminal balloon angioplasty and insertion of 3 or more drug-eluting stents into coronary artery |
| | K75.3 | Percutaneous transluminal balloon angioplasty and insertion of 1-2 stents into coronary artery |
| | K75.4 | Percutaneous transluminal balloon angioplasty and insertion of 3 or more stents into coronary artery NEC |
| | K75.8 | Other specified percutaneous transluminal balloon angioplasty and insertion of stent into coronary artery |
| | K75.9 | Unspecified percutaneous transluminal balloon angioplasty and insertion of stent into coronary artery |
| Implantation of cardiac pacemakers/defibrillators | K59 | Cardioverter defibrillator introduced through the vein |
| | K59.1 | Implantation of cardioverter defibrillator using one electrode lead |
| | K59.2 | Implantation of cardioverter defibrillator using two electrode leads |
| | K59.3 | Resiting of lead of cardioverter defibrillator |
| | K59.4 | Renewal of cardioverter defibrillator NEC |
| | K59.5 | Removal of cardioverter defibrillator |
| | K59.6 | Implantation of cardioverter defibrillator using three electrode leads |
| | K59.7 | Renewal of cardioverter defibrillator using three electrode leads |
| | K59.8 | Other specified cardioverter defibrillator introduced through the vein |
| | K59.9 | Unspecified cardioverter defibrillator introduced through the vein |

| | | |
|---|---|---|
| | K60 | Cardiac pacemaker system introduced through vein |
| | K60.1 | Implantation of intravenous cardiac pacemaker system NEC |
| | K60.2 | Resiting of lead of intravenous cardiac pacemaker system |
| | K60.3 | Renewal of intravenous cardiac pacemaker system NEC |
| | K60.4 | Removal of intravenous cardiac pacemaker system |
| | K60.5 | Implantation of intravenous single chamber cardiac pacemaker system |
| | K60.6 | Implantation of intravenous dual chamber cardiac pacemaker system |
| | K60.7 | Implantation of intravenous biventricular cardiac pacemaker system |
| | K60.8 | Other specified cardiac pacemaker system introduced through vein |
| | K60.9 | Unspecified cardiac pacemaker system introduced through vein |
| | K61 | Other cardiac pacemaker system |
| | K61.1 | Implantation of cardiac pacemaker system NEC |
| | K61.2 | Resiting of lead of cardiac pacemaker system NEC |
| | K61.3 | Renewal of cardiac pacemaker system NEC |
| | K61.4 | Removal of cardiac pacemaker system NEC |
| | K61.5 | Implantation of single chamber cardiac pacemaker system |
| | K61.6 | Implantation of dual chamber cardiac pacemaker system |
| | K61.7 | Implantation of biventricular cardiac pacemaker system |
| | K61.8 | Other specified other cardiac pacemaker system |
| | K61.9 | Unspecified other cardiac pacemaker system |
| | K73 | Other cardiac pacemaker system introduced through vein |
| | K73.1 | Renewal of intravenous single chamber cardiac pacemaker system |
| | K73.2 | Renewal of intravenous dual chamber cardiac pacemaker system |
| | K73.3 | Renewal of intravenous biventricular cardiac pacemaker |
| | K73.8 | Other specified other cardiac pacemaker system introduced through vein |
| | K73.9 | Unspecified other cardiac pacemaker system introduced through vein |
| Percutaneous valve procedures/heart catheterisation | K35 | Therapeutic transluminal operations on valve of heart |
| | K35.1 | Percutaneous transluminal mitral valvotomy |
| | K35.2 | Percutaneous transluminal aortic valvotomy |
| | K35.3 | Percutaneous transluminal tricuspid valvotomy |
| | K35.4 | Percutaneous transluminal pulmonary valvotomy |
| | K35.5 | Percutaneous transluminal valvuloplasty |
| | K35.6 | Percutaneous transluminal pulmonary valve perforation and dilation |
| | K35.7 | Percutaneous transluminal pulmonary valve replacement |
| | K35.8 | Other specified therapeutic transluminal operations on valve of heart |
| | K35.9 | Unspecified therapeutic transluminal operations on valve of heart |
| **GI Procedures** | | |
| Trans oesophageal echo (TOE) procedures | U20.2 | Transoesophageal echocardiography |
| Other oesophageal endoscopic procedures | G14 | Fibreoptic endoscopic extirpation of lesion of oesophagus |
| | G14.1 | Fibreoptic endoscopic snare resection of lesion of oesophagus |
| | G14.2 | Fibreoptic endoscopic laser destruction of lesion of oesophagus |
| | G14.3 | Fibreoptic endoscopic cauterisation of lesion of oesophagus |
| | G14.4 | Fibreoptic endoscopic injection sclerotherapy to varices of oesophagus |
| | G14.5 | Fibreoptic endoscopic destruction of lesion of oesophagus NEC |
| | G14.6 | Fibreoptic endoscopic submucosal resection of lesion of oesophagus |
| | G14.7 | Fibreoptic endoscopic photodynamic therapy of lesion of oesophagus |
| | G14.8 | Other specified fibreoptic endoscopic extirpation of lesion of oesophagus |
| | G14.9 | Unspecified fibreoptic endoscopic extirpation of lesion of oesophagus |
| | G15 | Other therapeutic fibreoptic endoscopic operations on oesophagus |
| | G15.1 | Fibreoptic endoscopic removal of foreign body from oesophagus |
| | G15.2 | Fibreoptic endoscopic balloon dilation of oesophagus |
| | G15.3 | Fibreoptic endoscopic dilation of oesophagus NEC |
| | G15.4 | Fibreoptic endoscopic insertion of tubal prosthesis into oesophagus |
| | G15.5 | Fibreoptic endoscopic dilation of web of oesophagus |
| | G15.6 | Fibreoptic endoscopic insertion of expanding metal stent into oesophagus NEC |
| | G15.7 | Fibreoptic endoscopic insertion of expanding covered metal stent into oesophagus |
| | G15.8 | Other specified other therapeutic fibreoptic endoscopic operations on oesophagus |



Thornhill MH, *et al. Heart* 2023; 109:223–231. doi: 10.1136/heartjnl-2022-321519

| | | |
|---|---|---|
| | G15.9 | Unspecified other therapeutic fibreoptic endoscopic operations on oesophagus |
| | G16 | Diagnostic fibreoptic endoscopic examination of oesophagus |
| | G16.1 | Diagnostic fibreoptic endoscopic examination of oesophagus and biopsy of lesion of oesophagus |
| | G16.2 | Diagnostic fibreoptic endoscopic ultrasound examination of oesophagus |
| | G16.3 | Diagnostic fibreoptic insertion of Bravo pH capsule into oesophagus |
| | G16.8 | Other specified diagnostic fibreoptic endoscopic examination of oesophagus |
| | G16.9 | Unspecified diagnostic fibreoptic endoscopic examination of oesophagus |
| | G17 | Endoscopic extirpation of lesion of oesophagus using rigid oesophagoscope |
| | G17.1 | Endoscopic snare resection of lesion of oesophagus using rigid oesophagoscope |
| | G17.2 | Endoscopic laser destruction of lesion of oesophagus using rigid oesophagoscope |
| | G17.3 | Endoscopic cauterisation of lesion of oesophagus using rigid oesophagoscope |
| | G17.4 | Endoscopic injection sclerotherapy to varices of oesophagus using rigid oesophagoscope |
| | G17.8 | Other specified endoscopic extirpation of lesion of oesophagus using rigid oesophagoscope |
| | G17.9 | Unspecified endoscopic extirpation of lesion of oesophagus using rigid oesophagoscope |
| | G18 | Other therapeutic endoscopic operations on oesophagus using rigid oesophagoscope |
| | G18.1 | Endoscopic removal of foreign body from oesophagus using rigid oesophagoscope |
| | G18.2 | Endoscopic balloon dilation of oesophagus using rigid oesophagoscope |
| | G18.3 | Endoscopic dilation of oesophagus using rigid oesophagoscope NEC |
| | G18.4 | Endoscopic insertion of tubal prosthesis into oesophagus using rigid oesophagoscope |
| | G18.5 | Dilation of web of oesophagus using rigid oesophagoscope |
| | G18.8 | Other specified other therapeutic endoscopic operations on oesophagus using rigid oesophagoscope |
| | G18.9 | Unspecified other therapeutic endoscopic operations on oesophagus using rigid oesophagoscope |
| | G19 | Diagnostic endoscopic examination of oesophagus using rigid oesophagoscope |
| | G19.1 | Diagnostic endoscopic examination of oesophagus and biopsy of lesion of oesophagus using rigid oesophagoscope |
| | G19.2 | Diagnostic endoscopic insertion of Bravo pH capsule using rigid oesophagoscope |
| | G19.8 | Other specified diagnostic endoscopic examination of oesophagus using rigid oesophagoscope |
| | G19.9 | Unspecified diagnostic endoscopic examination of oesophagus using rigid oesophagoscope |
| | G20 | Therapeutic fibreoptic endoscopic operations on oesophagus |
| | G20.1 | Fibreoptic endoscopic coagulation of bleeding lesion of oesophagus |
| | G20.8 | Other specified therapeutic fibreoptic endoscopic operations on oesophagus |
| | G20.9 | Unspecified therapeutic fibreoptic endoscopic operations on oesophagus |
| Upper GI endoscopic procedures (gastric, jejunum, ileum) | G42* | Other fibreoptic endoscopic extirpation of lesion of upper gastrointestinal tract |
| | G42.1* | Fibreoptic endoscopic submucosal resection of lesion of upper gastrointestinal tract |
| | G42.2* | Fibreoptic endoscopic photodynamic therapy of lesion of upper gastrointestinal tract |
| | G42.3* | Fibreoptic endoscopic mucosal resection of lesion of upper gastrointestinal tract |
| | G42.8* | Other specified other fibreoptic endoscopic extirpation of lesion of upper gastrointestinal tract |
| | G42.9* | Unspecified other fibreoptic endoscopic extirpation of lesion of upper gastrointestinal tract |
| | G43* | Fibreoptic endoscopic extirpation of lesion of upper gastrointestinal tract |
| | G43.1* | Fibreoptic endoscopic snare resection of lesion of upper gastrointestinal tract |
| | G43.2* | Fibreoptic endoscopic laser destruction of lesion of upper gastrointestinal tract |
| | G43.3* | Fibreoptic endoscopic cauterisation of lesion of upper gastrointestinal tract |
| | G43.4* | Fibreoptic endoscopic sclerotherapy to lesion of upper gastrointestinal tract |
| | G43.5* | Fibreoptic endoscopic destruction of lesion of upper gastrointestinal tract NEC |
| | G43.6* | Fibreoptic endoscopic injection therapy to lesion of upper gastrointestinal tract NEC |
| | G43.7* | Fibreoptic endoscopic rubber band ligation of upper gastrointestinal tract varices |

| | | |
|---|---|---|
| | G43.8* | Other specified fibreoptic endoscopic extirpation of lesion of upper gastrointestinal tract |
| | G43.9* | Unspecified fibreoptic endoscopic extirpation of lesion of upper gastrointestinal tract |
| | G44* | Other therapeutic fibreoptic endoscopic operations on upper gastrointestinal tract |
| | G44.1* | Fibreoptic endoscopic insertion of prosthesis into upper gastrointestinal tract |
| | G44.2# | Fibreoptic endoscopic removal of foreign body from upper gastrointestinal tract |
| | G44.3* | Fibreoptic endoscopic dilation of upper gastrointestinal tract NEC |
| | G44.4# | Fibreoptic endoscopic reduction of intussusception of gastroenterostomy |
| | G44.5* | Fibreoptic endoscopic percutaneous insertion of gastrostomy |
| | G44.6* | Fibreoptic endoscopic pressure controlled balloon dilation of lower oesophageal sphincter |
| | G44.7# | Fibreoptic endoscopic removal of gastrostomy tube |
| | G44.8* | Other specified other therapeutic fibreoptic endoscopic operations on upper gastrointestinal tract |
| | G44.9* | Unspecified other therapeutic fibreoptic endoscopic operations on upper gastrointestinal tract |
| | G45# | Diagnostic fibreoptic endoscopic examination of upper gastrointestinal tract |
| | G45.1* | Fibreoptic endoscopic examination of upper gastrointestinal tract and biopsy of lesion of upper gastrointestinal tract |
| | G45.2# | Fibreoptic endoscopic ultrasound examination of upper gastrointestinal tract |
| | G45.3# | Fibreoptic endoscopic insertion of Bravo pH capsule into upper gastrointestinal tract |
| | G45.4# | Fibreoptic endoscopic examination of upper gastrointestinal tract and staining of gastric mucosa |
| | G45.8# | Other specified diagnostic fibreoptic endoscopic examination of upper gastrointestinal tract |
| | G45.9# | Unspecified diagnostic fibreoptic endoscopic examination of upper gastrointestinal tract |
| | G46* | Therapeutic fibreoptic endoscopic operations on upper gastrointestinal tract |
| | G46.1* | Fibreoptic endoscopic endoluminal plication of gastro-oesophageal junction |
| | G46.2* | Fibreoptic endoscopic coagulation of bleeding lesion of upper gastrointestinal tract |
| | G46.8* | Other specified therapeutic fibreoptic endoscopic operations on upper gastrointestinal tract |
| | G46.9* | Unspecified therapeutic fibreoptic endoscopic operations on upper gastrointestinal tract |
| | G54* | Therapeutic endoscopic operations on duodenum |
| | G54.1* | Endoscopic extirpation of lesion of duodenum |
| | G54.2* | Endoscopic dilation of duodenum |
| | G54.3* | Endoscopic insertion of tubal prosthesis into duodenum |
| | G54.8* | Other specified therapeutic endoscopic operations on duodenum |
| | G54.9* | Unspecified therapeutic endoscopic operations on duodenum |
| | G55# | Diagnostic endoscopic examination of duodenum |
| | G55.1* | Diagnostic endoscopic examination of duodenum and biopsy of lesion of duodenum |
| | G55.8# | Other specified diagnostic endoscopic examination of duodenum |
| | G55.9# | Unspecified diagnostic endoscopic examination of duodenum |
| | G64* | Therapeutic endoscopic operations on jejunum |
| | G64.1* | Endoscopic extirpation of lesion of jejunum |
| | G64.2* | Endoscopic dilation of jejunum |
| | G64.3* | Endoscopic insertion of tubal prosthesis into jejunum |
| | G64.8* | Other specified therapeutic endoscopic operations on jejunum |
| | G64.9* | Unspecified therapeutic endoscopic operations on jejunum |
| | G65# | Diagnostic endoscopic examination of jejunum |
| | G65.1* | Diagnostic endoscopic examination of jejunum and biopsy of lesion of jejunum |
| | G65.8# | Other specified diagnostic endoscopic examination of jejunum |
| | G65.9# | Unspecified diagnostic endoscopic examination of jejunum |
| | G79* | Therapeutic endoscopic operations on ileum |
| | G79.1* | Endoscopic extirpation of lesion of ileum |
| | G79.2* | Endoscopic dilation of ileum |

| | | |
|---|---|---|
| | G79.3* | Endoscopic insertion of tubal prosthesis into ileum |
| | G79.8* | Other specified therapeutic endoscopic operations on ileum |
| | G79.9* | Unspecified therapeutic endoscopic operations on ileum |
| | G80# | Diagnostic endoscopic examination of ileum |
| | G80.1* | Diagnostic endoscopic examination of ileum and biopsy of lesion of ileum |
| | G80.2# | Wireless capsule endoscopy |
| | G80.3# | Diagnostic endoscopic balloon examination of ileum |
| | G80.8# | Other specified diagnostic endoscopic examination of ileum |
| | G80.9# | Unspecified diagnostic examination of ileum |
| Lower GI endoscopic procedures (including sigmoid and rectum) | H20* | Endoscopic extirpation of lesion of colon |
| | H20.1* | Fibreoptic endoscopic snare resection of lesion of colon |
| | H20.2* | Fibreoptic endoscopic cauterisation of lesion of colon |
| | H20.3* | Fibreoptic endoscopic laser destruction of lesion of colon |
| | H20.4* | Fibreoptic endoscopic destruction of lesion of colon NEC |
| | H20.5* | Fibreoptic endoscopic submucosal resection of lesion of colon |
| | H20.6* | Fibreoptic endoscopic resection of lesion of colon NEC |
| | H20.7* | Fibreoptic endoscopic mucosal resection of lesion of colon |
| | H20.8* | Other specified endoscopic extirpation of lesion of colon |
| | H20.9* | Unspecified endoscopic extirpation of lesion of colon |
| | H21* | Other therapeutic endoscopic operations on colon |
| | H21.1* | Fibreoptic endoscopic dilation of colon |
| | H21.2* | Fibreoptic endoscopic coagulation of blood vessel of colon |
| | H21.3# | Fibreoptic endoscopic removal of foreign body from colon |
| | H21.4* | Fibreoptic endoscopic insertion of expanding metal stent into colon |
| | H21.5# | Fibreoptic endoscopic decompression of colon |
| | H21.8* | Other specified other therapeutic endoscopic operations on colon |
| | H21.9* | Unspecified other therapeutic endoscopic operations on colon |
| | H22# | Diagnostic endoscopic examination of colon |
| | H22.1* | Diagnostic fibreoptic endoscopic examination of colon and biopsy of lesion of colon |
| | H22.8# | Other specified diagnostic endoscopic examination of colon |
| | H22.9# | Unspecified diagnostic endoscopic examination of colon |
| | H23* | Endoscopic extirpation of lesion of lower bowel using fibreoptic sigmoidoscope |
| | H23.1* | Endoscopic snare resection of lesion of lower bowel using fibreoptic sigmoidoscope |
| | H23.2* | Endoscopic cauterisation of lesion of lower bowel using fibreoptic sigmoidoscope |
| | H23.3* | Endoscopic laser destruction of lesion of lower bowel using fibreoptic sigmoidoscope |
| | H23.4* | Endoscopic destruction of lesion of lower bowel using fibreoptic sigmoidoscope NEC |
| | H23.5* | Endoscopic submucosal resection of lesion of lower bowel using fibreoptic sigmoidoscope |
| | H23.6* | Endoscopic resection of lesion of lower bowel using fibreoptic sigmoidoscope NEC |
| | H23.7* | Endoscopic mucosal resection of lesion of lower bowel using fibreoptic sigmoidoscope |
| | H23.8* | Other specified endoscopic extirpation of lesion of lower bowel using fibreoptic sigmoidoscope |
| | H23.9* | Unspecified endoscopic extirpation of lesion of lower bowel using fibreoptic sigmoidoscope |
| | H24* | Other therapeutic endoscopic operations on lower bowel using fibreoptic sigmoidoscope |
| | H24.1* | Endoscopic dilation of lower bowel using fibreoptic sigmoidoscope |
| | H24.2* | Endoscopic coagulation of blood vessel of lower bowel using fibreoptic sigmoidoscope |
| | H24.3* | Endoscopic insertion of tubal prosthesis into lower bowel using fibreoptic sigmoidoscope |
| | H24.4* | Endoscopic insertion of expanding metal stent into lower bowel using fibreoptic sigmoidoscope |
| | H24.5# | Endoscopic decompression of lower bowel using fibreoptic sigmoidoscope |
| | H24.8* | Other specified other therapeutic endoscopic operations on lower bowel using fibreoptic sigmoidoscope |

| | | |
|---|---|---|
| | H24.9* | Unspecified other therapeutic endoscopic operations on lower bowel using fibreoptic sigmoidoscope |
| | H25# | Diagnostic endoscopic examination of lower bowel using fibreoptic sigmoidoscope |
| | H25.1* | Diagnostic endoscopic examination of lower bowel and biopsy of lesion of lower bowel using fibreoptic sigmoidoscope |
| | H25.2# | Diagnostic endoscopic examination of lower bowel and sampling for bacterial overgrowth using fibreoptic sigmoidoscope |
| | H25.8# | Other specified diagnostic endoscopic examination of lower bowel using fibreoptic sigmoidoscope |
| | H25.9# | Unspecified diagnostic endoscopic examination of lower bowel using fibreoptic sigmoidoscope |
| | H26* | Endoscopic extirpation of lesion of sigmoid colon using rigid sigmoidoscope |
| | H26.1* | Endoscopic snare resection of lesion of sigmoid colon using rigid sigmoidoscope |
| | H26.2* | Endoscopic cauterisation of lesion of sigmoid colon using rigid sigmoidoscope |
| | H26.3* | Endoscopic laser destruction of lesion of sigmoid colon using rigid sigmoidoscope |
| | H26.4* | Endoscopic cryotherapy to lesion of sigmoid colon using rigid sigmoidoscope |
| | H26.5* | Endoscopic destruction of lesion of sigmoid colon using rigid sigmoidoscope NEC |
| | H26.6* | Endoscopic submucosal resection of lesion of sigmoid colon using rigid sigmoidoscope |
| | H26.7* | Endoscopic resection of lesion of sigmoid colon using rigid sigmoidoscope NEC |
| | H26.8* | Other specified endoscopic extirpation of lesion of sigmoid colon using rigid sigmoidoscope |
| | H26.9* | Unspecified endoscopic extirpation of lesion of sigmoid colon using rigid sigmoidoscope |
| | H27* | Other therapeutic endoscopic operations on sigmoid colon using rigid sigmoidoscope |
| | H27.1* | Endoscopic dilation of sigmoid colon using rigid sigmoidoscope |
| | H27.2# | Endoscopic removal of foreign body from sigmoid colon using rigid sigmoidoscope |
| | H27.3* | Endoscopic insertion of tubal prosthesis into sigmoid colon using rigid sigmoidoscope |
| | H27.4* | Endoscopic insertion of expanding metal stent into sigmoid colon using rigid sigmoidoscope |
| | H27.5# | Endoscopic decompression of sigmoid colon using rigid sigmoidoscope |
| | H27.8* | Other specified other therapeutic endoscopic operations on sigmoid colon using rigid sigmoidoscope |
| | H27.9* | Unspecified other therapeutic endoscopic operations on sigmoid colon using rigid sigmoidoscope |
| | H28# | Diagnostic endoscopic examination of sigmoid colon using rigid sigmoidoscope |
| | H28.1* | Diagnostic endoscopic examination of sigmoid colon and biopsy of lesion of sigmoid colon using rigid sigmoidoscope |
| | H28.8# | Other specified diagnostic endoscopic examination of sigmoid colon using rigid sigmoidoscope |
| | H28.9# | Unspecified diagnostic endoscopic examination of sigmoid colon using rigid sigmoidoscope |
| Colonic surgery (incl. appendix) | H01 | Emergency excision of appendix |
| | H01.1 | Emergency excision of abnormal appendix and drainage HFQ |
| | H01.2 | Emergency excision of abnormal appendix NEC |
| | H01.3 | Emergency excision of normal appendix |
| | H01.8 | Other specified emergency excision of appendix |
| | H01.9 | Unspecified emergency excision of appendix |
| | H02 | Other excision of appendix |
| | H02.1 | Interval appendicectomy |
| | H02.2 | Planned delayed appendicectomy NEC |
| | H02.3 | Prophylactic appendicectomy NEC |
| | H02.4 | Incidental appendicectomy |
| | H02.8 | Other specified other excision of appendix |
| | H02.9 | Unspecified other excision of appendix |
| | H03 | Other operations on appendix |

| | | |
|---|---|---|
| | H03.1 | Drainage of abscess of appendix |
| | H03.2 | Drainage of appendix NEC |
| | H03.3 | Exteriorisation of appendix |
| | H03.8 | Other specified other operations on appendix |
| | H03.9 | Unspecified other operations on appendix |
| | H04 | Total excision of colon and rectum |
| | H04.1 | Panproctocolectomy and ileostomy |
| | H04.2 | Panproctocolectomy and anastomosis of ileum to anus and creation of pouch HFQ |
| | H04.3 | Panproctocolectomy and anastomosis of ileum to anus NEC |
| | H04.8 | Other specified total excision of colon and rectum |
| | H04.9 | Unspecified total excision of colon and rectum |
| | H05 | Total excision of colon |
| | H05.1 | Total colectomy and anastomosis of ileum to rectum |
| | H05.2 | Total colectomy and ileostomy and creation of rectal fistula HFQ |
| | H05.3 | Total colectomy and ileostomy NEC |
| | H05.8 | Other specified total excision of colon |
| | H05.9 | Unspecified total excision of colon |
| | H06 | Extended excision of right hemicolon |
| | H06.1 | Extended right hemicolectomy and end to end anastomosis |
| | H06.2 | Extended right hemicolectomy and anastomosis of ileum to colon |
| | H06.3 | Extended right hemicolectomy and anastomosis NEC |
| | H06.4 | Extended right hemicolectomy and ileostomy HFQ |
| | H06.5 | Extended right hemicolectomy and end to side anastomosis |
| | H06.8 | Other specified extended excision of right hemicolon |
| | H06.9 | Unspecified extended excision of right hemicolon |
| | H07 | Other excision of right hemicolon |
| | H07.1 | Right hemicolectomy and end to end anastomosis of ileum to colon |
| | H07.2 | Right hemicolectomy and side to side anastomosis of ileum to transverse colon |
| | H07.3 | Right hemicolectomy and anastomosis NEC |
| | H07.4 | Right hemicolectomy and ileostomy HFQ |
| | H07.5 | Right hemicolectomy and end to side anastomosis |
| | H07.8 | Other specified other excision of right hemicolon |
| | H07.9 | Unspecified other excision of right hemicolon |
| | H08 | Excision of transverse colon |
| | H08.1 | Transverse colectomy and end to end anastomosis |
| | H08.2 | Transverse colectomy and anastomosis of ileum to colon |
| | H08.3 | Transverse colectomy and anastomosis NEC |
| | H08.4 | Transverse colectomy and ileostomy HFQ |
| | H08.5 | Transverse colectomy and exteriorisation of bowel NEC |
| | H08.6 | Transverse colectomy and end to side anastomosis |
| | H08.8 | Other specified excision of transverse colon |
| | H08.9 | Unspecified excision of transverse colon |
| | H09 | Excision of left hemicolon |
| | H09.1 | Left hemicolectomy and end to end anastomosis of colon to rectum |
| | H09.2 | Left hemicolectomy and end to end anastomosis of colon to colon |
| | H09.3 | Left hemicolectomy and anastomosis NEC |
| | H09.4 | Left hemicolectomy and ileostomy HFQ |
| | H09.5 | Left hemicolectomy and exteriorisation of bowel NEC |
| | H09.6 | Left hemicolectomy and end to side anastomosis |
| | H09.8 | Other specified excision of left hemicolon |
| | H09.9 | Unspecified excision of left hemicolon |
| | H10 | Excision of sigmoid colon |
| | H10.1 | Sigmoid colectomy and end to end anastomosis of ileum to rectum |
| | H10.2 | Sigmoid colectomy and anastomosis of colon to rectum |
| | H10.3 | Sigmoid colectomy and anastomosis NEC |
| | H10.4 | Sigmoid colectomy and ileostomy HFQ |
| | H10.5 | Sigmoid colectomy and exteriorisation of bowel NEC |
| | H10.6 | Sigmoid colectomy and end to side anastomosis |

| | | |
|---|---|---|
| | H10.8 | Other specified excision of sigmoid colon |
| | H10.9 | Unspecified excision of sigmoid colon |
| | H11 | Other excision of colon |
| | H11.1 | Colectomy and end to end anastomosis of colon to colon NEC |
| | H11.2 | Colectomy and side to side anastomosis of ileum to colon NEC |
| | H11.3 | Colectomy and anastomosis NEC |
| | H11.4 | Colectomy and ileostomy NEC |
| | H11.5 | Colectomy and exteriorisation of bowel NEC |
| | H11.6 | Colectomy and end to side anastomosis NEC |
| | H11.8 | Other specified other excision of colon |
| | H11.9 | Unspecified other excision of colon |
| | H12 | Extirpation of lesion of colon |
| | H12.1 | Excision of diverticulum of colon |
| | H12.2 | Excision of lesion of colon NEC |
| | H12.3 | Destruction of lesion of colon NEC |
| | H12.8 | Other specified extirpation of lesion of colon |
| | H12.9 | Unspecified extirpation of lesion of colon |
| | H13 | Bypass of colon |
| | H13.1 | Bypass of colon by anastomosis of ileum to colon |
| | H13.2 | Bypass of colon by anastomosis of caecum to sigmoid colon |
| | H13.3 | Bypass of colon by anastomosis of transverse colon to sigmoid colon |
| | H13.4 | Bypass of colon by anastomosis of transverse colon to rectum |
| | H13.5 | Bypass of colon by anastomosis of colon to rectum NEC |
| | H13.8 | Other specified bypass of colon |
| | H13.9 | Unspecified bypass of colon |
| | H14 | Exteriorisation of caecum |
| | H14.1 | Tube caecostomy |
| | H14.2 | Refashioning of caecostomy |
| | H14.3 | Closure of caecostomy |
| | H14.4 | Appendicocaecostomy |
| | H14.8 | Other specified exteriorisation of caecum |
| | H14.9 | Unspecified exteriorisation of caecum |
| | H15 | Other exteriorisation of colon |
| | H15.1 | Loop colostomy |
| | H15.2 | End colostomy |
| | H15.3 | Refashioning of colostomy |
| | H15.4 | Closure of colostomy |
| | H15.5 | Dilation of colostomy |
| | H15.6 | Reduction of prolapse of colostomy |
| | H15.7 | Percutaneous endoscopic sigmoid colostomy |
| | H15.8 | Other specified other exteriorisation of colon |
| | H15.9 | Unspecified other exteriorisation of colon |
| | H16 | Incision of colon |
| | H16.1 | Drainage of colon |
| | H16.2 | Caecotomy |
| | H16.3 | Colotomy |
| | H16.8 | Other specified incision of colon |
| | H16.9 | Unspecified incision of colon |
| | H17 | Intra-abdominal manipulation of colon |
| | H17.1 | Open reduction of intussusception of colon |
| | H17.2 | Open reduction of volvulus of caecum |
| | H17.3 | Open reduction of volvulus of sigmoid colon |
| | H17.4 | Open reduction of volvulus of colon NEC |
| | H17.5 | Open relief of strangulation of colon |
| | H17.6 | Open relief of obstruction of colon NEC |
| | H17.8 | Other specified intra-abdominal manipulation of colon |
| | H17.9 | Unspecified intra-abdominal manipulation of colon |
| | H18 | Open endoscopic operations on colon |

| | | |
|---|---|---|
| | H18.1 | Open colonoscopy |
| | H18.8 | Other specified open endoscopic operations on colon |
| | H18.9 | Unspecified open endoscopic operations on colon |
| | H19 | Other open operations on colon |
| | H19.1 | Open biopsy of lesion of colon |
| | H19.2 | Fixation of colon |
| | H19.3 | Enterorrhaphy of colon |
| | H19.4 | Open removal of foreign body from colon |
| | H19.8 | Other specified other open operations on colon |
| | H19.9 | Unspecified other open operations on colon |
| | H29 | Subtotal excision of colon |
| | H29.1 | Subtotal excision of colon and rectum and creation of colonic pouch and anastomosis of colon to anus |
| | H29.2 | Subtotal excision of colon and rectum and creation of colonic pouch NEC |
| | H29.3 | Subtotal excision of colon and creation of colonic pouch and anastomosis of colon to rectum |
| | H29.4 | Subtotal excision of colon and creation of colonic pouch NEC |
| | H29.5 | Subtotal excision of colon and anastomosis of colon to ileum |
| | H29.8 | Other specified subtotal excision of colon |
| | H29.9 | Unspecified subtotal excision of colon |
| Endoscopic Retrograde Cholangio-pancreatic Procedures (ERCP) | J40 | Endoscopic retrograde placement of prosthesis in bile duct |
| | J40.1 | Endoscopic retrograde insertion of tubal prosthesis into both hepatic ducts |
| | J40.2 | Endoscopic retrograde insertion of tubal prosthesis into bile duct NEC |
| | J40.3 | Endoscopic retrograde renewal of tubal prosthesis in bile duct NEC |
| | J40.4 | Endoscopic retrograde removal of tubal prosthesis from bile duct |
| | J40.5 | Endoscopic retrograde insertion of expanding covered metal stent into bile duct |
| | J40.6 | Endoscopic retrograde insertion of expanding metal stent into bile duct NEC |
| | J40.7 | Endoscopic retrograde renewal of expanding metal stent in bile duct |
| | J40.8 | Other specified endoscopic retrograde placement of prosthesis in bile duct |
| | J40.9 | Unspecified endoscopic retrograde placement of prosthesis in bile duct |
| | J41 | Other therapeutic endoscopic retrograde operations on bile duct |
| | J41.1 | Endoscopic retrograde extraction of calculus from bile duct |
| | J41.2 | Endoscopic dilation of bile duct NEC |
| | J41.3 | Endoscopic retrograde lithotripsy of calculus of bile duct |
| | J41.4 | Endoscopic retrograde photodynamic laser therapy of lesion of bile duct |
| | J41.8 | Other specified other therapeutic endoscopic retrograde operations on bile duct |
| | J41.9 | Unspecified other therapeutic endoscopic retrograde operations on bile duct |
| | J42 | Therapeutic endoscopic retrograde operations on pancreatic duct |
| | J42.1 | Endoscopic retrograde insertion of tubal prosthesis into pancreatic duct |
| | J42.2 | Endoscopic retrograde renewal of tubal prosthesis in pancreatic duct |
| | J42.3 | Endoscopic retrograde removal of calculus from pancreatic duct |
| | J42.4 | Endoscopic retrograde drainage of lesion of pancreas |
| | J42.5 | Endoscopic retrograde dilation of pancreatic duct |
| | J42.8 | Other specified therapeutic endoscopic retrograde operations on pancreatic duct |
| | J42.9 | Unspecified therapeutic endoscopic retrograde operations on pancreatic duct |
| | J43 | Diagnostic endoscopic retrograde examination of bile duct and pancreatic duct |
| | J43.1 | Endoscopic retrograde cholangiopancreatography and biopsy of lesion of ampulla of Vater |
| | J43.2 | Endoscopic retrograde cholangiopancreatography and biopsy of lesion of biliary or pancreatic system NEC |
| | J43.3 | Endoscopic retrograde cholangiopancreatography and collection of bile |
| | J43.8 | Other specified diagnostic endoscopic retrograde examination of bile duct and pancreatic duct |
| | J43.9 | Unspecified diagnostic endoscopic retrograde examination of bile duct and pancreatic duct |
| | J44 | Diagnostic endoscopic retrograde examination of bile duct |
| | J44.1 | Endoscopic retrograde cholangiography and biopsy of lesion of bile duct |
| | J44.8 | Other specified diagnostic endoscopic retrograde examination of bile duct |

| | | |
|---|---|---|
| | J44.9 | Unspecified diagnostic endoscopic retrograde examination of bile duct |
| | J45 | Diagnostic endoscopic retrograde examination of pancreatic duct |
| | J45.1 | Endoscopic retrograde pancreatography and biopsy of lesion of pancreas |
| | J45.2 | Endoscopic retrograde pancreatography and collection of pancreatic juice |
| | J45.3 | Endoscopic retrograde pancreatography through accessory ampulla of Vater |
| | J45.8 | Other specified diagnostic endoscopic retrograde examination of pancreatic duct |
| | J45.9 | Unspecified diagnostic endoscopic retrograde examination of pancreatic duct |
| **GU Procedures** | | |
| Cystoscopic procedures | M09 | Therapeutic endoscopic operations on calculus of kidney |
| | M09.1 | Endoscopic ultrasound fragmentation of calculus of kidney |
| | M09.2 | Endoscopic electrohydraulic shockwave fragmentation of calculus of kidney |
| | M09.3 | Endoscopic laser fragmentation of calculus of kidney |
| | M09.4 | Endoscopic extraction of calculus of kidney NEC |
| | M09.8 | Other specified therapeutic endoscopic operations on calculus of kidney |
| | M09.9 | Unspecified therapeutic endoscopic operations on calculus of kidney |
| | M10 | Other therapeutic endoscopic operations on kidney |
| | M10.1 | Endoscopic extirpation of lesion of kidney NEC |
| | M10.2 | Endoscopic pyeloplasty |
| | M10.3 | Endoscopic deroofing of multiple cysts of kidney |
| | M10.4 | Endoscopic cryoablation of lesion of kidney |
| | M10.5 | Endoscopic endoluminal balloon rupture of stenosis of pelviureteric junction of kidney |
| | M10.8 | Other specified other therapeutic endoscopic operations on kidney |
| | M10.9 | Unspecified other therapeutic endoscopic operations on kidney |
| | M11 | Diagnostic endoscopic examination of kidney |
| | M11.1 | Diagnostic endoscopic examination of kidney and biopsy of lesion of kidney NEC |
| | M11.2 | Diagnostic endoscopic retrograde examination of kidney and biopsy of lesion of kidney |
| | M11.3 | Diagnostic endoscopic retrograde examination of kidney NEC |
| | M11.8 | Other specified diagnostic endoscopic examination of kidney |
| | M11.9 | Unspecified diagnostic endoscopic examination of kidney |
| | M27 | Therapeutic ureteroscopic operations on ureter |
| | M27.1 | Ureteroscopic laser fragmentation of calculus of ureter |
| | M27.2 | Ureteroscopic fragmentation of calculus of ureter NEC |
| | M27.3 | Ureteroscopic extraction of calculus of ureter |
| | M27.4 | Ureteroscopic insertion of ureteric stent |
| | M27.5 | Ureteroscopic removal of ureteric stent |
| | M27.6 | Ureteroscopic endoluminal balloon rupture of stenosis of ureter |
| | M27.7 | Ureteroscopic dilation of ureter |
| | M27.8 | Other specified therapeutic ureteroscopic operations on ureter |
| | M27.9 | Unspecified therapeutic ureteroscopic operations on ureter |
| | M28 | Other endoscopic removal of calculus from ureter |
| | M28.1 | Code retired - refer to introduction |
| | M28.2 | Code retired - refer to introduction |
| | M28.3 | Code retired - refer to introduction |
| | M28.4 | Endoscopic catheter drainage of calculus of ureter |
| | M28.5 | Endoscopic drainage of calculus of ureter by dilation of ureter |
| | M28.8 | Other specified other endoscopic removal of calculus from ureter |
| | M28.9 | Unspecified other endoscopic removal of calculus from ureter |
| | M29 | Other therapeutic endoscopic operations on ureter |
| | M29.1 | Endoscopic extirpation of lesion of ureter |
| | M29.2 | Endoscopic insertion of tubal prosthesis into ureter NEC |
| | M29.3 | Endoscopic removal of tubal prosthesis from ureter |
| | M29.4 | Endoscopic dilation of ureter |
| | M29.5 | Endoscopic renewal of tubal prosthesis into ureter |
| | M29.8 | Other specified other therapeutic endoscopic operations on ureter |
| | M29.9 | Unspecified other therapeutic endoscopic operations on ureter |

| | | |
|---|---|---|
| | M30 | Diagnostic endoscopic examination of ureter |
| | M30.1 | Endoscopic retrograde pyelography |
| | M30.2 | Endoscopic catheterisation of ureter |
| | M30.3 | Endoscopic ureteric urine sampling |
| | M30.4 | Nephroscopic ureteroscopy |
| | M30.5 | Diagnostic endoscopic examination of ureter and biopsy of lesion of ureter NEC |
| | M30.6 | Diagnostic endoscopic examination of ureter and biopsy of lesion of ureter using rigid ureteroscope |
| | M30.8 | Other specified diagnostic endoscopic examination of ureter |
| | M30.9 | Unspecified diagnostic endoscopic examination of ureter |
| | M42 | Endoscopic extirpation of lesion of bladder |
| | M42.1 | Endoscopic resection of lesion of bladder |
| | M42.2 | Endoscopic cauterisation of lesion of bladder |
| | M42.3 | Endoscopic destruction of lesion of bladder NEC |
| | M42.8 | Other specified endoscopic extirpation of lesion of bladder |
| | M42.9 | Unspecified endoscopic extirpation of lesion of bladder |
| | M43 | Endoscopic operations to increase capacity of bladder |
| | M43.1 | Endoscopic transection of bladder |
| | M43.2 | Endoscopic hydrostatic distension of bladder |
| | M43.3 | Endoscopic overdistension of bladder NEC |
| | M43.4 | Endoscopic injection of neurolytic substance into nerve of bladder |
| | M43.8 | Other specified endoscopic operations to increase capacity of bladder |
| | M43.9 | Unspecified endoscopic operations to increase capacity of bladder |
| | M44 | Other therapeutic endoscopic operations on bladder |
| | M44.1 | Endoscopic lithopaxy |
| | M44.2 | Endoscopic extraction of calculus of bladder NEC |
| | M44.3 | Endoscopic removal of foreign body from bladder |
| | M44.4 | Endoscopic removal of blood clot from bladder |
| | M44.8 | Other specified other therapeutic endoscopic operations on bladder |
| | M44.9 | Unspecified other therapeutic endoscopic operations on bladder |
| | M45 | Diagnostic endoscopic examination of bladder |
| | M45.1 | Diagnostic endoscopic examination of bladder and biopsy of lesion of bladder NEC |
| | M45.2 | Diagnostic endoscopic examination of bladder and biopsy of lesion of prostate NEC |
| | M45.3 | Diagnostic endoscopic examination of bladder and biopsy of lesion of bladder using rigid cystoscope |
| | M45.4 | Diagnostic endoscopic examination of bladder and biopsy of lesion of prostate using rigid cystoscope |
| | M45.5 | Diagnostic endoscopic examination of bladder using rigid cystoscope |
| | M45.8 | Other specified diagnostic endoscopic examination of bladder |
| | M45.9 | Unspecified diagnostic endoscopic examination of bladder |
| Endoscopic prostate procedures | M65 | Endoscopic resection of outlet of male bladder |
| | M65.1 | Endoscopic resection of prostate using electrotome |
| | M65.2 | Endoscopic resection of prostate using punch |
| | M65.3 | Endoscopic resection of prostate NEC |
| | M65.4 | Endoscopic resection of prostate using laser |
| | M65.5 | Endoscopic resection of prostate using vapotrode |
| | M65.6 | Endoscopic ablation of prostate using steam |
| | M65.8 | Other specified endoscopic resection of outlet of male bladder |
| | M65.9 | Unspecified endoscopic resection of outlet of male bladder |
| | M66 | Other therapeutic endoscopic operations on outlet of male bladder |
| | M66.1 | Endoscopic sphincterotomy of external sphincter of male bladder |
| | M66.2 | Endoscopic incision of outlet of male bladder NEC |
| | M66.3 | Endoscopic injection of inert substance into outlet of male bladder |
| | M66.8 | Other specified other therapeutic endoscopic operations on outlet of male bladder |
| | M66.9 | Unspecified other therapeutic endoscopic operations on outlet of male bladder |
| | M67 | Other therapeutic endoscopic operations on prostate |
| | M67.1 | Endoscopic cryotherapy to lesion of prostate |
| | M67.2 | Endoscopic destruction of lesion of prostate NEC |

| | | |
|---|---|---|
| | M67.3 | Endoscopic drainage of prostate |
| | M67.4 | Endoscopic removal of calculus from prostate |
| | M67.5 | Endoscopic microwave destruction of lesion of prostate |
| | M67.6 | Endoscopic radiofrequency ablation of lesion of prostate |
| | M67.8 | Other specified other therapeutic endoscopic operations on prostate |
| | M67.9 | Unspecified other therapeutic endoscopic operations on prostate |
| | M68 | Endoscopic insertion of prosthesis into prostate |
| | M68.1 | Endoscopic insertion of prostatic stent |
| | M68.2 | Endoscopic removal of prostatic stent |
| | M68.3 | Endoscopic insertion of prosthesis to compress lobe of prostate |
| | M68.8 | Other specified endoscopic insertion of prosthesis into prostate |
| | M68.9 | Unspecified endoscopic insertion of prosthesis into prostate |
| | M70.1 | Aspiration of prostate NEC |
| | M70.2 | Perineal needle biopsy of prostate |
| | M70.3 | Rectal needle biopsy of prostate |
| | M70.4 | Balloon dilation of prostate |
| | M70.5 | Massage of prostate |
| | M70.6 | Radioactive seed implantation into prostate |
| | M70.7 | Transurethral radiofrequency needle ablation of prostate |
| | M70.8 | Other specified other operations on outlet of male bladder |
| | M70.9 | Unspecified other operations on outlet of male bladder |
| | M71 | Other operations on prostate |
| | M71.1 | High intensity focused ultrasound of prostate |
| | M71.2 | Implantation of radioactive substance into prostate |
| | M71.8 | Other specified other operations on prostate |
| | M71.9 | Unspecified other operations on prostate |
| **Respiratory Procedures** | | |
| Bronchoscopic procedures | E48 | Therapeutic fibreoptic endoscopic operations on lower respiratory tract |
| | E48.1 | Fibreoptic endoscopic snare resection of lesion of lower respiratory tract |
| | E48.2 | Fibreoptic endoscopic laser destruction of lesion of lower respiratory tract |
| | E48.3 | Fibreoptic endoscopic destruction of lesion of lower respiratory tract NEC |
| | E48.4 | Fibreoptic endoscopic aspiration of lower respiratory tract |
| | E48.5 | Fibreoptic endoscopic removal of foreign body from lower respiratory tract |
| | E48.6 | Fibreoptic endoscopic irrigation of lower respiratory tract |
| | E48.7 | Fibreoptic endoscopic photodynamic therapy of lesion of lower respiratory tract |
| | E48.8 | Other specified therapeutic fibreoptic endoscopic operations on lower respiratory tract |
| | E48.9 | Unspecified therapeutic fibreoptic endoscopic operations on lower respiratory tract |
| | E49 | Diagnostic fibreoptic endoscopic examination of lower respiratory tract |
| | E49.1 | Diagnostic fibreoptic endoscopic examination of lower respiratory tract and biopsy of lesion of lower respiratory tract |
| | E49.2 | Diagnostic fibreoptic endoscopic examination of lower respiratory tract and lavage of lesion of lower respiratory tract |
| | E49.3 | Diagnostic fibreoptic endoscopic examination of lower respiratory tract and brush cytology of lesion of lower respiratory tract |
| | E49.4 | Diagnostic fibreoptic endoscopic examination of lower respiratory tract with lavage and brush cytology of lesion of lower respiratory tract |
| | E49.5 | Diagnostic fibreoptic endoscopic examination of lower respiratory tract with biopsy, lavage and brush cytology of lesion of lower respiratory tract |
| | E49.8 | Other specified diagnostic fibreoptic endoscopic examination of lower respiratory tract |
| | E49.9 | Unspecified diagnostic fibreoptic endoscopic examination of lower respiratory tract |
| | E50 | Therapeutic endoscopic operations on lower respiratory tract using rigid bronchoscope |
| | E50.1 | Endoscopic snare resection of lesion of lower respiratory tract using rigid bronchoscope |
| | E50.2 | Endoscopic laser destruction of lesion of lower respiratory tract using rigid bronchoscope |



Thornhill MH, *et al. Heart* 2023; 109:223–231. doi: 10.1136/heartjnl-2022-321519

| | | |
|---|---|---|
| | E50.3 | Endoscopic destruction of lesion of lower respiratory tract using rigid bronchoscope NEC |
| | E50.4 | Endoscopic aspiration of lower respiratory tract using rigid bronchoscope |
| | E50.5 | Endoscopic removal of foreign body from lower respiratory tract using rigid bronchoscope |
| | E50.6 | Endoscopic irrigation of lower respiratory tract using rigid bronchoscope |
| | E50.8 | Other specified therapeutic endoscopic operations on lower respiratory tract using rigid bronchoscope |
| | E50.9 | Unspecified therapeutic endoscopic operations on lower respiratory tract using rigid bronchoscope |
| | E51 | Diagnostic endoscopic examination of lower respiratory tract using rigid bronchoscope |
| | E51.1 | Diagnostic endoscopic examination of lower respiratory tract and biopsy of lesion of lower respiratory tract using rigid bronchoscope |
| | E51.8 | Other specified diagnostic endoscopic examination of lower respiratory tract using rigid bronchoscope |
| | E51.9 | Unspecified diagnostic endoscopic examination of lower respiratory tract using rigid bronchoscope |
| **ENT procedures** | | |
| Tonsillectomy & Adenoidectomy | E20 | Operations on adenoid |
| | E20.1 | Total adenoidectomy |
| | E20.2 | Biopsy of adenoid |
| | E20.3 | Surgical arrest of postoperative bleeding of adenoid |
| | E20.4 | Suction diathermy adenoidectomy |
| | E20.8 | Other specified operations on adenoid |
| | E20.9 | Unspecified operations on adenoid |
| | F34 | Excision of tonsil |
| | F34.1 | Bilateral dissection tonsillectomy |
| | F34.2 | Bilateral guillotine tonsillectomy |
| | F34.3 | Bilateral laser tonsillectomy |
| | F34.4 | Bilateral excision of tonsil NEC |
| | F34.5 | Excision of remnant of tonsil |
| | F34.6 | Excision of lingual tonsil |
| | F34.7 | Bilateral coblation tonsillectomy |
| | F34.8 | Other specified excision of tonsil |
| | F34.9 | Unspecified excision of tonsil |
| | F36 | Other operations on tonsil |
| | F36.1 | Destruction of tonsil |
| | F36.2 | Biopsy of lesion of tonsil |
| | F36.3 | Drainage of abscess of peritonsillar region |
| | F36.4 | Removal of foreign body from tonsil |
| | F36.5 | Surgical arrest of postoperative bleeding from tonsillar bed |
| | F36.6 | Excision of lesion of tonsil |
| | F36.8 | Other specified other operations on tonsil |
| | F36.9 | Unspecified other operations on tonsil |
| Nasal packing/nasal intubation | E06 | Packing of cavity of nose |
| | E06.1 | Packing of posterior cavity of nose NEC |
| | E06.2 | Packing of anterior cavity of nose NEC |
| | E06.3 | Removal of packing from cavity of nose |
| | E06.4 | Balloon packing of cavity of nose |
| | E06.8 | Other specified packing of cavity of nose |
| | E06.9 | Unspecified packing of cavity of nose |
| | X56.1 | Nasotracheal intubation |
| **Haematology Procedures** | | |
| Blood transfusion/red cell or plasma exchange | X32 | Exchange blood transfusion |
| | X32.1 | Neonatal exchange blood transfusion |
| | X32.2 | Exchange of plasma (single) |

| | | |
|---|---|---|
| | X32.3 | Exchange of plasma (2-9) |
| | X32.4 | Exchange of plasma (10-19) |
| | X32.5 | Exchange of plasma (>19) |
| | X32.6 | Red cell exchange |
| | X32.7 | Leucopheresis |
| | X32.8 | Other specified exchange blood transfusion |
| | X32.9 | Unspecified exchange blood transfusion |
| | X33 | Other blood transfusion |
| | X33.1 | Intra-arterial blood transfusion |
| | X33.2 | Intravenous blood transfusion of packed cells |
| | X33.3 | Intravenous blood transfusion of platelets |
| | X33.4 | Autologous peripheral blood stem cell transplant |
| | X33.5 | Syngeneic peripheral blood stem cell transplant |
| | X33.6 | Allogeneic peripheral blood stem cell transplant |
| | X33.7 | Autologous transfusion of red blood cells |
| | X33.8 | Other specified other blood transfusion |
| | X33.9 | Unspecified other blood transfusion |
| | X34 | Other intravenous transfusion |
| | X34.1 | Transfusion of coagulation factor |
| | X34.2 | Transfusion of plasma NEC |
| | X34.3 | Transfusion of serum NEC |
| | X34.4 | Transfusion of blood expander |
| | X34.8 | Other specified other intravenous transfusion |
| | X34.9 | Unspecified other intravenous transfusion |
| Bone marrow puncture | W36.5 | Diagnostic extraction of bone marrow NEC |
| | Y66.7 | Harvest of bone marrow |
| **Obstetric & gynae procedures** | | |
| Abortion/dilatation & curettage | Q10 | Curettage of uterus |
| | Q10.1 | Dilation of cervix uteri and curettage of products of conception from uterus |
| | Q10.2 | Curettage of products of conception from uterus NEC |
| | Q10.3 | Dilation of cervix uteri and curettage of uterus NEC |
| | Q10.8 | Other specified curettage of uterus |
| | Q10.9 | Unspecified curettage of uterus |
| | Q11 | Other evacuation of contents of uterus |
| | Q11.1 | Vacuum aspiration of products of conception from uterus NEC |
| | Q11.2 | Dilation of cervix uteri and evacuation of products of conception from uterus NEC |
| | Q11.3 | Evacuation of products of conception from uterus NEC |
| | Q11.4 | Extraction of menses |
| | Q11.5 | Vacuum aspiration of products of conception from uterus using rigid cannula |
| | Q11.6 | Vacuum aspiration of products of conception from uterus using flexible cannula |
| | Q11.8 | Other specified other evacuation of contents of uterus |
| | Q11.9 | Unspecified other evacuation of contents of uterus |
| Vaginal delivery | R19 | Breech extraction delivery |
| | R19.1 | Breech extraction delivery with version |
| | R19.8 | Other specified breech extraction delivery |
| | R19.9 | Unspecified breech extraction delivery |
| | R20 | Other breech delivery |
| | R20.1 | Spontaneous breech delivery |
| | R20.2 | Assisted breech delivery |
| | R20.8 | Other specified other breech delivery |
| | R20.9 | Unspecified other breech delivery |
| | R21 | Forceps cephalic delivery |
| | R21.1 | High forceps cephalic delivery with rotation |
| | R21.2 | High forceps cephalic delivery NEC |
| | R21.3 | Mid forceps cephalic delivery with rotation |
| | R21.4 | Mid forceps cephalic delivery NEC |
| | R21.5 | Low forceps cephalic delivery |

| | | |
|---|---|---|
| | R21.8 | Other specified forceps cephalic delivery |
| | R21.9 | Unspecified forceps cephalic delivery |
| | R22 | Vacuum delivery |
| | R22.1 | High vacuum delivery |
| | R22.2 | Low vacuum delivery |
| | R22.3 | Vacuum delivery before full dilation of cervix |
| | R22.8 | Other specified vacuum delivery |
| | R22.9 | Unspecified vacuum delivery |
| | R23 | Cephalic vaginal delivery with abnormal presentation of head at delivery without instrument |
| | R23.1 | Manipulative cephalic vaginal delivery with abnormal presentation of head at delivery without instrument |
| | R23.2 | Non-manipulative cephalic vaginal delivery with abnormal presentation of head at delivery without instrument |
| | R23.8 | Other specified cephalic vaginal delivery with abnormal presentation of head at delivery without instrument |
| | R23.9 | Unspecified cephalic vaginal delivery with abnormal presentation of head at delivery without instrument |
| | R24 | Normal delivery |
| | R24.9 | All normal delivery |
| Caesarean delivery | R17 | Elective caesarean delivery |
| | R17.1 | Elective upper uterine segment caesarean delivery |
| | R17.2 | Elective lower uterine segment caesarean delivery |
| | R17.8 | Other specified elective caesarean delivery |
| | R17.9 | Unspecified elective caesarean delivery |
| | R18 | Other caesarean delivery |
| | R18.1 | Upper uterine segment caesarean delivery NEC |
| | R18.2 | Lower uterine segment caesarean delivery NEC |
| | R18.8 | Other specified other caesarean delivery |
| | R18.9 | Unspecified other caesarean delivery |
| **Skin Procedures** | | |
| Skin and wound management procedures | S41 | Suture of skin of head or neck |
| | S41.1 | Primary suture of skin of head or neck NEC |
| | S41.2 | Delayed primary suture of skin of head or neck |
| | S41.3 | Secondary suture of skin of head or neck |
| | S41.4 | Resuture of skin of head or neck |
| | S41.8 | Other specified suture of skin of head or neck |
| | S41.9 | Unspecified suture of skin of head or neck |
| | S42 | Suture of skin of other site |
| | S42.1 | Primary suture of skin NEC |
| | S42.2 | Delayed primary suture of skin NEC |
| | S42.3 | Secondary suture of skin NEC |
| | S42.4 | Resuture of skin NEC |
| | S42.8 | Other specified suture of skin of other site |
| | S42.9 | Unspecified suture of skin of other site |
| | S47 | Opening of skin |
| | S47.1 | Drainage of lesion of skin of head or neck |
| | S47.2 | Drainage of lesion of skin NEC |
| | S47.3 | Incision of lesion of skin of head or neck |
| | S47.4 | Incision of lesion of skin NEC |
| | S47.5 | Incision of skin of head or neck |
| | S47.6 | Incision of skin NEC |
| | S47.8 | Other specified opening of skin |
| | S47.9 | Unspecified opening of skin |
| | S54 | Exploration of burnt skin of head or neck |
| | S54.1 | Debridement of burnt skin of head or neck |
| | S54.2 | Removal of slough from burnt skin of head or neck |
| | S54.3 | Toilet to burnt skin of head or neck NEC |

| | | |
|---|---|---|
| | S54.4 | Dressing of burnt skin of head or neck NEC |
| | S54.5 | Attention to dressing of burnt skin of head or neck |
| | S54.6 | Cleansing and sterilisation of burnt skin of head or neck |
| | S54.7 | Dressing of burnt skin of head or neck using vacuum assisted closure device |
| | S54.8 | Other specified exploration of burnt skin of head or neck |
| | S54.9 | Unspecified exploration of burnt skin of head or neck |
| | S55 | Exploration of burnt skin of other site |
| | S55.1 | Debridement of burnt skin NEC |
| | S55.2 | Removal of slough from burnt skin NEC |
| | S55.3 | Toilet to burnt skin NEC |
| | S55.4 | Dressing of burnt skin NEC |
| | S55.5 | Attention to dressing of burnt skin NEC |
| | S55.6 | Cleansing and sterilisation of burnt skin NEC |
| | S55.7 | Dressing of burnt skin using vacuum assisted closure device NEC |
| | S55.8 | Other specified exploration of burnt skin of other site |
| | S55.9 | Unspecified exploration of burnt skin of other site |
| | S56 | Exploration of other skin of head or neck |
| | S56.1 | Debridement of skin of head or neck NEC |
| | S56.2 | Removal of slough from skin of head or neck NEC |
| | S56.3 | Toilet to skin of head or neck NEC |
| | S56.4 | Dressing of skin of head or neck NEC |
| | S56.5 | Attention to dressing of skin of head or neck NEC |
| | S56.6 | Cleansing and sterilisation of skin of head or neck NEC |
| | S56.7 | Dressing of skin of head or neck using vacuum assisted closure device NEC |
| | S56.8 | Other specified exploration of other skin of head or neck |
| | S56.9 | Unspecified exploration of other skin of head or neck |
| | S57 | Exploration of other skin of other site |
| | S57.1 | Debridement of skin NEC |
| | S57.2 | Removal of slough from skin NEC |
| | S57.3 | Toilet of skin NEC |
| | S57.4 | Dressing of skin NEC |
| | S57.5 | Attention to dressing of skin NEC |
| | S57.6 | Cleansing and sterilisation of skin NEC |
| | S57.7 | Dressing of skin using vacuum assisted closure device NEC |
| | S57.8 | Other specified exploration of other skin of other site |
| | S57.9 | Unspecified exploration of other skin of other site |
| **Dental Procedures** | | |
| Extractions & surgical removal of teeth | F09 | Surgical removal of tooth |
| | F09.1 | Surgical removal of impacted wisdom tooth |
| | F09.2 | Surgical removal of impacted tooth NEC |
| | F09.3 | Surgical removal of wisdom tooth NEC |
| | F09.4 | Surgical removal of tooth NEC |
| | F09.5 | Surgical removal of retained root of tooth |
| | F09.8 | Other specified surgical removal of tooth |
| | F09.9 | Unspecified surgical removal of tooth |
| | F10 | Simple extraction of tooth |
| | F10.1 | Full dental clearance |
| | F10.2 | Upper dental clearance |
| | F10.3 | Lower dental clearance |
| | F10.4 | Extraction of multiple teeth NEC |
| | F10.8 | Other specified simple extraction of tooth |
| | F10.9 | Unspecified simple extraction of tooth |
| Other oral surgical procedures | F01 | Partial excision of lip |
| | F01.1 | Excision of vermilion border of lip and advancement of mucosa of lip |
| | F01.8 | Other specified partial excision of lip |
| | F01.9 | Unspecified partial excision of lip |
| | F02 | Extirpation of lesion of lip |
| | F02.1 | Excision of lesion of lip |

| | | |
|---|---|---|
| | F02.2 | Destruction of lesion of lip |
| | F02.8 | Other specified extirpation of lesion of lip |
| | F02.9 | Unspecified extirpation of lesion of lip |
| | F03 | Correction of deformity of lip |
| | F03.1 | Primary closure of cleft lip |
| | F03.2 | Revision of primary closure of cleft lip |
| | F03.3 | Adjustment to vermilion border of lip NEC |
| | F03.8 | Other specified correction of deformity of lip |
| | F03.9 | Unspecified correction of deformity of lip |
| | F04 | Other reconstruction of lip |
| | F04.1 | Reconstruction of lip using tongue flap |
| | F04.2 | Reconstruction of lip using skin flap |
| | F04.8 | Other specified other reconstruction of lip |
| | F04.9 | Unspecified other reconstruction of lip |
| | F05 | Other repair of lip |
| | F05.1 | Excision of excess mucosa from lip |
| | F05.2 | Advancement of mucosa of lip NEC |
| | F05.3 | Suture of lip |
| | F05.4 | Removal of suture from lip |
| | F05.8 | Other specified other repair of lip |
| | F05.9 | Unspecified other repair of lip |
| | F06 | Other operations on lip |
| | F06.1 | Division of adhesions of lip |
| | F06.2 | Biopsy of lesion of lip |
| | F06.3 | Shave of lip |
| | F06.8 | Other specified other operations on lip |
| | F06.9 | Unspecified other operations on lip |
| | F08 | Implantation of tooth |
| | F08.1 | Allotransplantation of tooth |
| | F08.2 | Autotransplantation of tooth |
| | F08.3 | Replantation of tooth |
| | F08.4 | Repositioning of tooth |
| | F08.8 | Other specified implantation of tooth |
| | F08.9 | Unspecified implantation of tooth |
| | F11 | Preprosthetic oral surgery |
| | F11.1 | Oral alveoplasty |
| | F11.2 | Augmentation of alveolar ridge using autobone graft |
| | F11.3 | Augmentation of alveolar ridge NEC |
| | F11.4 | Vestibuloplasty of mouth |
| | F11.5 | Endosseous implantation into jaw |
| | F11.6 | Subperiosteal implantation into jaw |
| | F11.8 | Other specified preprosthetic oral surgery |
| | F11.9 | Unspecified preprosthetic oral surgery |
| | F18 | Excision of dental lesion of jaw |
| | F18.1 | Enucleation of dental cyst of jaw |
| | F18.2 | Marsupialisation of dental lesion of jaw |
| | F18.8 | Other specified excision of dental lesion of jaw |
| | F18.9 | Unspecified excision of dental lesion of jaw |
| | F22 | Excision of tongue |
| | F22.1 | Total glossectomy |
| | F22.2 | Partial glossectomy |
| | F22.8 | Other specified excision of tongue |
| | F22.9 | Unspecified excision of tongue |
| | F23 | Extirpation of lesion of tongue |
| | F23.1 | Excision of lesion of tongue |
| | F23.2 | Destruction of lesion of tongue |
| | F23.8 | Other specified extirpation of lesion of tongue |
| | F23.9 | Unspecified extirpation of lesion of tongue |

| | | |
|---|---|---|
| | F24 | Incision of tongue |
| | F24.1 | Biopsy of lesion of tongue |
| | F24.2 | Removal of foreign body from tongue |
| | F24.3 | Glossotomy |
| | F24.8 | Other specified incision of tongue |
| | F24.9 | Unspecified incision of tongue |
| | F26 | Other operations on tongue |
| | F26.1 | Commissurectomy of tongue |
| | F26.2 | Excision of frenulum of tongue |
| | F26.3 | Incision of frenulum of tongue |
| | F26.4 | Freeing of adhesions of tongue |
| | F26.5 | Suture of tongue |
| | F26.8 | Other specified other operations on tongue |
| | F26.9 | Unspecified other operations on tongue |
| | F28 | Extirpation of lesions of palate |
| | F28.1 | Excision of lesion of palate |
| | F28.2 | Destruction of lesion of palate |
| | F28.8 | Other specified extirpation of lesion of palate |
| | F28.9 | Unspecified extirpation of lesion of palate |
| | F29 | Correction of deformity of palate |
| | F29.1 | Primary repair of cleft palate |
| | F29.2 | Revision of repair of cleft palate |
| | F29.8 | Other specified correction of deformity of cleft palate |
| | F29.9 | Unspecified correction of deformity of palate |
| | F30 | Other repair of palate |
| | F30.1 | Plastic repair of palate using flap of palate |
| | F30.2 | Plastic repair of palate using flap of skin |
| | F30.3 | Plastic repair of palate using flap of tongue |
| | F30.4 | Plastic repair of palate using graft of skin |
| | F30.5 | Plastic repair of palate using flap of mucosa |
| | F30.6 | Plastic repair of palate using graft of mucosa |
| | F30.7 | Suture of palate |
| | F30.8 | Other specified other repair of palate |
| | F30.9 | Unspecified other repair of palate |
| | F32 | Other operations on palate |
| | F32.1 | Biopsy of lesion of palate |
| | F32.2 | Removal of foreign body from palate |
| | F32.3 | Incision of palate |
| | F32.4 | Operations on uvula NEC |
| | F32.5 | Uvulopalatopharyngoplasty |
| | F32.6 | Uvulopalatoplasty |
| | F32.8 | Other specified other operations on palate |
| | F32.9 | Unspecified other operations on palate |
| | F38 | Extirpation of lesion of other part of mouth |
| | F38.1 | Excision of lesion of floor of mouth |
| | F38.2 | Excision of lesion of mouth NEC |
| | F38.3 | Destruction of lesion of floor of mouth |
| | F38.4 | Destruction of lesion of mouth NEC |
| | F38.8 | Other specified extirpation of lesion of other part of mouth |
| | F38.9 | Unspecified extirpation of lesion of other part of mouth |
| | F39 | Reconstruction of other part of mouth |
| | F39.1 | Reonstruction of mouth using flap NEC |
| | F39.2 | Reconstruction of mouth using graft NEC |
| | F39.8 | other specified reconstruction of other part of mouth |
| | F39.9 | Unspecified reconstruction of other part of mouth |
| | F40 | Other repair of other part of mouth |
| | F40.1 | Revision of repair of mouth NEC |
| | F40.2 | Graft of skin to mouth NEC |



Thornhill MH, *et al. Heart* 2023; 109:223–231. doi: 10.1136/heartjnl-2022-321519

| | | |
|---|---|---|
| | F40.3 | Graft of mucosa to mouth NEC |
| | F40.4 | Suture of mouth NEC |
| | F40.5 | Removal of suture from mouth NEC |
| | F40.8 | Other specified other repair of other part of mouth |
| | F40.9 | Unspecified other repair of other part of mouth |
| | F42.1 | Biopsy of lesion of mouth NEC |
| | F42.2 | Incision of mouth NEC |
| | F42.3 | Removal of excess mucosa from mouth NEC |
| Endodontic procedures | F12 | Surgery on apex of tooth |
| | F12.1 | Apicectomy of tooth |
| | F12.2 | Root canal therapy to tooth |
| | F12.8 | Other specified surgery on apex of tooth |
| | F12.9 | Unspecified surgery on apex of tooth |
| Scaling and gingival procedures | F16.4 | Scaling of tooth |
| | F20 | Operations on gingiva |
| | F20.1 | Excision of gingiva |
| | F20.2 | Excision of lesion of gingiva |
| | F20.3 | Biopsy of lesion of gingiva |
| | F20.4 | Gingivoplasty |
| | F20.5 | Suture of gingiva |
| | F20.8 | Other specified operations on gingiva |
| | F20.9 | Unspecified operations on gingiva |
| Restorative Dental Procedures | F13.1 | Full restoration of crown of tooth |
| | F13.2 | Partial restoration of crown of tooth |
| | F13.3 | Restoration of crown of tooth NEC |
| | F13.4 | Restoration of part of using inlay NEC |
| | F13.5 | Restoration of part of tooth using filling NEC |
| | F13.8 | Other specified restoration of tooth |
| | F13.9 | Unspecified restoration of tooth |
| | F17.1 | Preparation of tooth for dental crown |
| | F17.6 | Preparation of teeth for bridge |

Note: * = endoscopic GI procedures treated as interventional in sub analysis of upper and lower GI endoscopic procedures, # = endoscopic GI procedures treated as non-interventional

**Table S6.** Case-crossover step-analysis comparing the incidence of invasive procedures (IPs) using a 4-month case period and preceding 11-month control period for 14,731 patients admitted with IE.

| Invasive Procedures (IPs) | Case Period (4m) | | Control Period (11m) | | Unadjusted step model[b] | | Adjusted step-model[c] | | |
|---|---|---|---|---|---|---|---|---|---|
| | Total proc[a] | Proc/m[a] | Total proc[a] | Proc/m[a] | OR | 95% CI | OR | 95% CI | p-value |
| **Cardiac Procedures** | | | | | | | | | |
| Coronary angiography | 465 | 116.3 | 892 | 81.1 | 1.44 | 1.28 to 1.61 | 0.97 | 0.81 to 1.16 | 0.897 |
| Coronary artery bypass graft (CABG) | 15 | 3.8 | 9 | 0.8 | 4.58 | 2.01 to 10.48 | 6.31 | 1.14 to 34.48 | 0.156 |
| Percutaneous coronary procedures and stents | 65 | 16.3 | 86 | 7.8 | 2.1 | 1.52 to 2.90 | 1.42 | 0.81 to 2.49 | 0.497 |
| Implantation of cardiac pacemakers/defibrillators | 547 | 136.8 | 588 | 53.5 | 2.6 | 2.32 to 2.93 | 1.29 | 1.05 to 1.58 | 0.086 |
| Percutaneous valve procedures | 9 | 2.3 | 29 | 2.6 | 0.85 | 0.40 to 1.81 | 1.15 | 0.35 to 3.72 | 0.895 |
| | | | | | | | | | |
| **Dental Procedures** | | | | | | | | | |
| Extractions or surgical removal of teeth | 69 | 17.3 | 66 | 6 | 2.89 | 2.06 to 4.05 | 3.35 | 1.75 to 6.39 | 0.021 |
| Other oral surgical procedures | 14 | 3.5 | 24 | 2.2 | 1.61 | 0.83 to 3.13 | 0.84 | 0.29 to 2.46 | 0.865 |
| Scaling and gingival procedures | 1 | 0.3 | 1 | 0.1 | 2.75 | 0.17 to 43.90 | 1.06 | 0.01 to 126.76 | 0.981 |
| | | | | | | | | | |
| **ENT procedures** | | | | | | | | | |
| Tonsillectomy & Adenoidectomy | 3 | 0.8 | 5 | 0.5 | 1.65 | 0.39 to 6.90 | 0.31 | 0.03 to 2.95 | 0.588 |
| Nasal packing/nasal intubation | 33 | 8.3 | 49 | 4.5 | 1.93 | 1.22 to 3.04 | 1.03 | 0.48 to 2.02 | 0.979 |
| | | | | | | | | | |
| **GI Procedures** | | | | | | | | | |
| Upper GI endoscopic procedures (gastric, duodenum, jejunum, ileum) | 754 | 188.5 | 887 | 80.6 | 2.43 | 2.20 to 2.69 | 1.43 | 1.20 to 1.69 | 0.007 |
| Lower GI endoscopic procedures (including sigmoid and rectum) | 412 | 103 | 577 | 52.5 | 2 | 1.76 to 2.27 | 1.69 | 1.35 to 2.12 | 0.010 |
| Colonic surgery (incl appendix) | 33 | 8.3 | 54 | 4.9 | 1.68 | 1.09 to 2.59 | 1.15 | 0.56 to 2.36 | 0.910 |
| Endoscopic Retrograde Cholangio-pancreatic Procedures (ERCP) | 35 | 8.8 | 47 | 4.3 | 2.13 | 1.36 to 3.35 | 1.42 | 0.65 to 3.07 | 0.577 |
| | | | | | | | | | |
| **GU Procedures** | | | | | | | | | |
| Cystoscopic procedures | 204 | 51 | 452 | 41.1 | 1.27 | 1.07 to 1.51 | 0.9 | 0.69 to 1.19 | 0.670 |
| Endoscopic prostate procedures | 53 | 13.3 | 93 | 8.5 | 1.57 | 1.12 to 2.21 | 0.59 | 0.35 to 1.02 | 0.222 |



Thornhill MH, *et al. Heart* 2023; 109:223–231. doi: 10.1136/heartjnl-2022-321519

| | | | | | | | | | |
|---|---|---|---|---|---|---|---|---|---|
| | | | | | | | | | |
| **Haematology Procedures** | | | | | | | | | |
| Blood transfusion/red cell or plasma exchange | 368 | 92 | 587 | 53.4 | 2.7 | 2.26 to 3.24 | 1.15 | 0.86 to 1.54 | 0.633 |
| Bone marrow puncture | 114 | 28.5 | 129 | 11.7 | 2.58 | 1.99 to 3.35 | 1.29 | 0.82 to 2.02 | 0.562 |
| | | | | | | | | | |
| **Obstetric & gynaecological procedures** | | | | | | | | | |
| Abortion/dilatation & curettage | 5 | 1.3 | 10 | 0.9 | 1.38 | 0.47 to 4.07 | 1.66 | 0.27 to 10.32 | 0.797 |
| Vaginal delivery | 8 | 2 | 28 | 2.5 | 0.79 | 0.36 to 1.72 | 0.59 | 0.19 to 1.86 | 0.611 |
| Caesarean delivery | 5 | 1.3 | 7 | 0.6 | 1.96 | 0.62 to 6.19 | 10.86 | 0.84 to 140.64 | 0.223 |
| | | | | | | | | | |
| **Respiratory Procedures** | | | | | | | | | |
| Bronchoscopic procedures | 60 | 15 | 63 | 5.7 | 2.65 | 1.86 to 3.79 | 1.63 | 0.87 to 3.05 | 0.374 |
| | | | | | | | | | |
| **Skin Procedures** | | | | | | | | | |
| Skin and wound management procedures | 134 | 33.5 | 268 | 24.4 | 1.39 | 1.12 to 1.71 | 0.78 | 0.56 to 1.09 | 0.355 |

Notes: a, a maximum of one procedure of each type per patient was counted each month. b, period-adjusted OR of IPs in case period (4 months prior to IE admission) compared to control period (15 to 5 months prior to IE admission) calculated using a mixed effects logistic regression model with the patient as the random effect. c, OR of IPs in case period (4 months prior to IE admission) compared to control period (15 to 5 months prior to IE admission) calculated using a mixed effects logistic regression model adjusted for month and date of IE admission with the patient as the random effect. m=month, proc=procedures, OR=odds ratio, CI=confidence interval, IE=infective endocarditis, IPs=invasive procedures. P values in red = significant positive association between the IP and subsequent IE following Benjamini-Hochberg correction. P values in purple = significant negative association between IP and subsequent IE following Benjamini-Hochberg correction.



Thornhill MH, *et al. Heart* 2023; 109:223–231. doi: 10.1136/heartjnl-2022-321519

**Table S7.** Case-crossover step-analysis comparing the incidence of invasive procedures (IPs) using a 6-month case period and preceding 9-month control period for 14,731 patients admitted with IE.

| Invasive Procedures (IPs) | Case Period (4m) | | Control Period (11m) | | Unadjusted step model[b] | | Adjusted step-model[c] | | |
|---|---|---|---|---|---|---|---|---|---|
| | Total proc[a] | Proc/m[a] | Total proc[a] | Proc/m[a] | OR | 95% CI | OR | 95% CI | p-value |
| **Cardiac Procedures** | | | | | | | | | |
| Coronary angiography | 656 | 109.3 | 701 | 77.9 | 1.41 | 1.26 to 1.57 | 0.86 | 0.70 to 1.06 | 0.909 |
| Coronary artery bypass graft (CABG) | 15 | 2.5 | 9 | 1 | 2.5 | 1.09 to 5.72 | 0.62 | 0.12 to 3.12 | 0.806 |
| Percutaneous coronary procedures and stents | 81 | 13.5 | 70 | 7.8 | 1.75 | 1.27 to 2.41 | 0.74 | 0.40 to 1.39 | 1.000 |
| Implantation of cardiac pacemakers/defibrillators | 702 | 117 | 433 | 48.1 | 2.47 | 2.19 to 2.79 | 0.98 | 0.78 to 1.24 | 0.878 |
| Percutaneous valve procedures | 12 | 2 | 26 | 2.9 | 069 | 0.34 to 1.37 | 0.59 | 0.17 to 2.04 | 0.718 |
| | | | | | | | | | |
| **Dental Procedures** | | | | | | | | | |
| Extractions or surgical removal of teeth | 80 | 13.3 | 55 | 6.1 | 2.19 | 1.55 to 3.09 | 1.37 | 0.69 to 2.69 | 0.940 |
| Other oral surgical procedures | 20 | 3.3 | 18 | 2 | 1.68 | 0.88 to 3.18 | 0.86 | 0.25 to 2.99 | 0.930 |
| Scaling and gingival procedures | 1 | 0.2 | 1 | 0.1 | 1.5 | 0.09 to 23.94 | 0.08 | 0.00 to 27.91 | 0.755 |
| | | | | | | | | | |
| **ENT procedures** | | | | | | | | | |
| Tonsillectomy & Adenoidectomy | 5 | 0.8 | 3 | 0.3 | 2.5 | 0.60 to 10.46 | 0.77 | 0.05 to 12.62 | 0.894 |
| Nasal packing/nasal intubation | 43 | 7.2 | 39 | 4.3 | 1.71 | 1.09 to 2.67 | 0.61 | 0.26 to 1.45 | 0.874 |
| | | | | | | | | | |
| **GI Procedures** | | | | | | | | | |
| Upper GI endoscopic procedures (gastric, duodenum, jejunum, ileum) | 972 | 162 | 669 | 74.3 | 2.25 | 2.04 to 2.49 | 1.07 | 0.88 to 1.31 | 0.747 |
| Lower GI Endoscopic procedures (including sigmoid and rectum) | 510 | 85 | 479 | 53.2 | 1.62 | 1.42 to 1.83 | 0.86 | 0.67 to 1.09 | 0.980 |
| Colonic surgery (incl appendix) | 43 | 7.2 | 44 | 4.9 | 1.47 | 0.96 to 2.23 | 0.69 | 0.31 to 1.56 | 0.860 |
| Endoscopic Retrograde Cholangio-pancreatic Procedures (ERCP) | 46 | 7.7 | 36 | 4 | 1.98 | 1.27 to 3.10 | 1.13 | 0.47 to 2.73 | 0.947 |
| | | | | | | | | | |
| **GU Procedures** | | | | | | | | | |
| Cystoscopic procedures | 313 | 52.2 | 343 | 38.1 | 1.41 | 1.20 to 1.66 | 1.27 | 0.93 to 1.74 | 1.000 |
| Endoscopic prostate procedures | 86 | 14.3 | 60 | 6.7 | 2.17 | 1.55 to 3.02 | 1.48 | 0.77 to 2.85 | 0.924 |



Thornhill MH, *et al. Heart* 2023; 109:223–231. doi: 10.1136/heartjnl-2022-321519

| | | | | | | | | | |
|---|---|---|---|---|---|---|---|---|---|
| **Haematology Procedures** | | | | | | | | | |
| Blood transfusion/red cell or plasma exchange | 503 | 83.8 | 452 | 50.2 | 2.54 | 2.13 to 3.02 | 0.88 | 0.63 to 1.23 | 0.731 |
| Bone marrow puncture | 149 | 24.8 | 94 | 10.4 | 2.51 | 1.92 to 3.27 | 1.1 | 0.65 to 1.86 | 0.912 |
| | | | | | | | | | |
| **Obstetric & gynaecological procedures** | | | | | | | | | |
| Abortion/dilatation & curettage | 6 | 1 | 9 | 1 | 1 | 0.35 to 2.83 | 0.62 | 0.09 to 4.29 | 0.846 |
| Vaginal delivery | 11 | 1.8 | 25 | 2.8 | 0.66 | 0.32 to 1.34 | 0.26 | 0.08 to 0.91 | 0.805 |
| Caesarean delivery | 6 | 1 | 6 | 0.7 | 1.5 | 0.48 to 4.65 | 6.15 | 0.51 to 74.18 | 1.000 |
| | | | | | | | | | |
| **Respiratory Procedures** | | | | | | | | | |
| Bronchoscopic procedures | 74 | 12.3 | 49 | 5.4 | 2.29 | 1.59 to 3.29 | 0.93 | 0.46 to 1.90 | 0.932 |
| | | | | | | | | | |
| **Skin Procedures** | | | | | | | | | |
| Skin and wound management procedures | 207 | 34.5 | 195 | 21.7 | 1.61 | 1.32 to 1.96 | 1.19 | 0.80 to 1.75 | 0.815 |

Notes: a, a maximum of one procedure of each type per patient was counted each month. b, period-adjusted OR of IPs in case period (6 months prior to IE admission) compared to control period (15 to 7 months prior to IE admission) calculated using a mixed effects logistic regression model with the patient as the random effect. c, OR of IPs in case period (6 months prior to IE admission) compared to control period (15 to 7 months prior to IE admission) calculated using a mixed effects logistic regression model adjusted for month and date of IE admission with the patient as the random effect. m=month, proc=procedures, OR=odds ratio, CI=confidence interval, IE=infective endocarditis, IPs=invasive procedures. P values in red = significant positive association between the IP and subsequent IE following Benjamini-Hochberg correction. P values in purple = significant negative association between IP and subsequent IE following Benjamini-Hochberg correction.

**Figure S1.** Incidence of different invasive procedures (IPs) over the 15 months before infective endocarditis (IE) hospital admission.

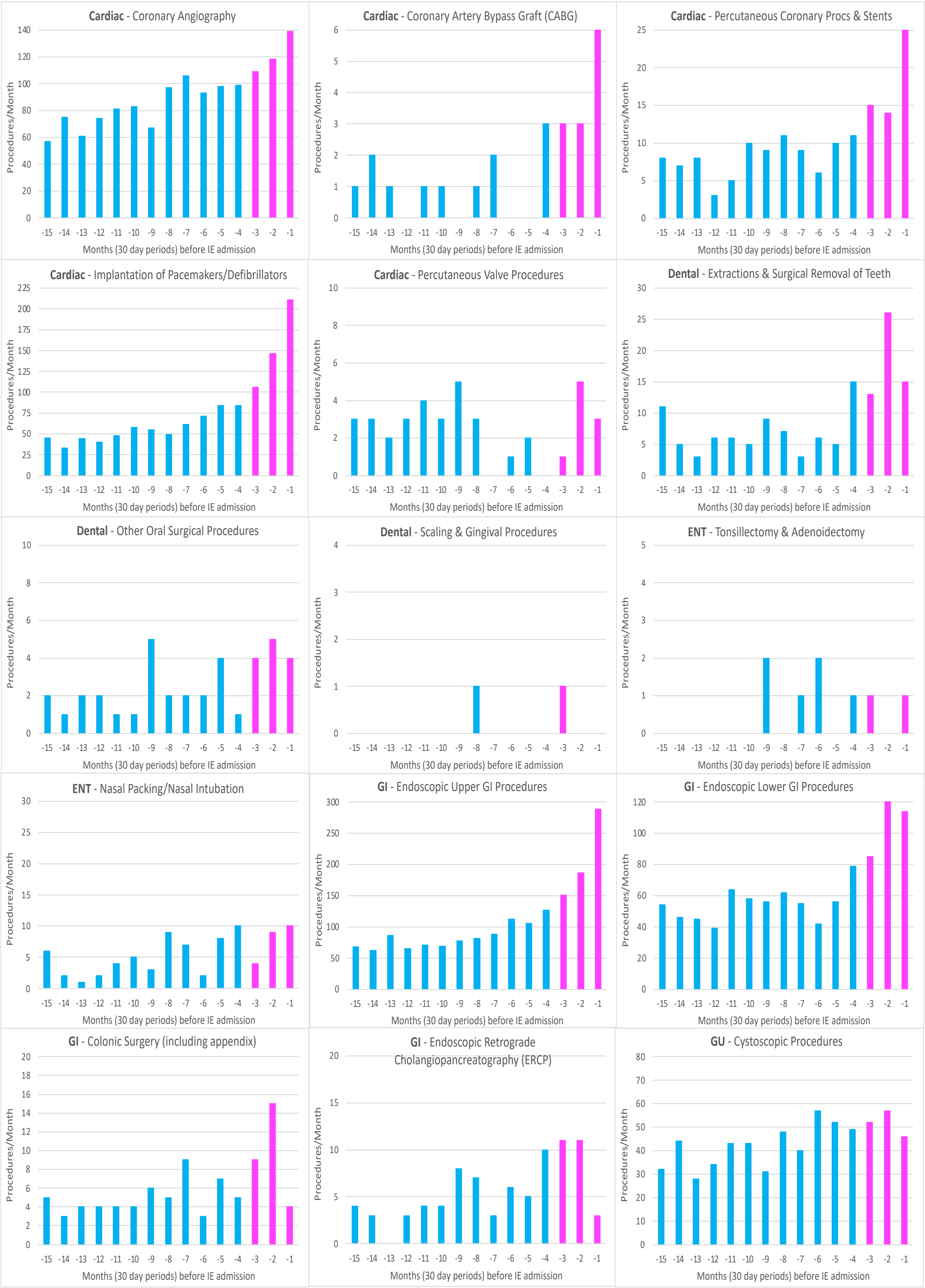




Thornhill MH, *et al. Heart* 2023; 109:223–231. doi: 10.1136/heartjnl-2022-321519

**Figure S1.** Continued.

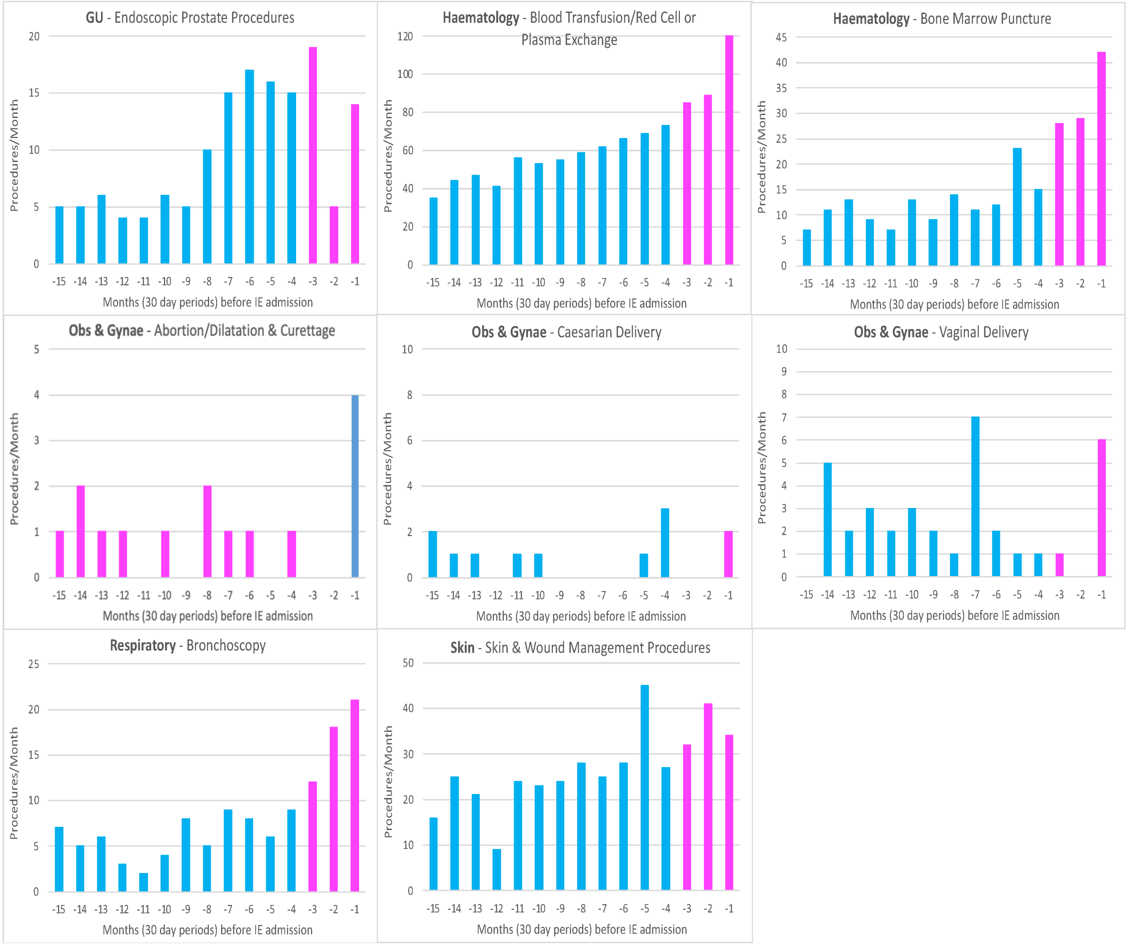


Notes: ENT = Ear, nose and throat, GI = gastrointestinal, GU = genitourinary, IE = infective endocarditis, Obs & Gynae = Obstetrics and gynaecology, Procs = procedures. Cyan bars represent the control-period (months -4 to -15). Magenta bars represent the case-period (months -1 to -3), IE admission - day zero.



Thornhill MH, *et al. Heart* 2023; 109:223–231. doi: 10.1136/heartjnl-2022-321519

## References:

1 Ramsdale DR, Turner-Stokes L, Advisory Group of the British Cardiac Society Clinical Practice C, et al. Prophylaxis and treatment of infective endocarditis in adults: a concise guide. *Clin Med (Lond)* 2004;**4**:545-50.

2 Horstkotte D, Follath F, Gutschik E, et al. Guidelines on prevention, diagnosis and treatment of infective endocarditis executive summary; the task force on infective endocarditis of the European society of cardiology. *Eur Heart J* 2004;**25**:267-76.

3 Dajani AS, Taubert KA, Wilson W, et al. Prevention of bacterial endocarditis. Recommendations by the American Heart Association. *JAMA* 1997;**277**:1794-801.

4 Janszky I, Gemes K, Ahnve S, et al. Invasive Procedures Associated With the Development of Infective Endocarditis. *J Am Coll Cardiol* 2018;**71**:2744-52.

5 Habib G, Hoen B, Tornos P, et al. Guidelines on the prevention, diagnosis, and treatment of infective endocarditis (new version 2009): the Task Force on the Prevention, Diagnosis, and Treatment of Infective Endocarditis of the European Society of Cardiology (ESC). Endorsed by the European Society of Clinical Microbiology and Infectious Diseases (ESCMID) and the International Society of Chemotherapy (ISC) for Infection and Cancer. *Eur Heart J* 2009;**30**:2369-413.

6 Wilson W, Taubert KA, Gewitz M, et al. Prevention of infective endocarditis: guidelines from the American Heart Association: a guideline from the American Heart Association Rheumatic Fever, Endocarditis, and Kawasaki Disease Committee, Council on Cardiovascular Disease in the Young, and the Council on Clinical Cardiology, Council on Cardiovascular Surgery and Anesthesia, and the Quality of Care and Outcomes Research Interdisciplinary Working Group. *Circulation* 2007;**116**:1736-54.

7 National Institute for Health and Care Excellence (NICE). Prophylaxis against infective endocarditis. Clinical Guideline [CG64]. National Institute for Health and Care Excellence (NICE) 2008:NICE Clinical Guideline No 64.



Thornhill MH, *et al. Heart* 2023; 109:223–231. doi: 10.1136/heartjnl-2022-321519
